# Mind the Data Gap: Using a Multi‐Measurement Synthesis for Identifying the Challenges and Opportunities in Studying Plant Drought Response and Recovery

**DOI:** 10.1111/pce.15349

**Published:** 2025-01-14

**Authors:** Jean V. Wilkening, Todd E. Dawson, Sally E. Thompson

**Affiliations:** ^1^ Civil and Environmental Engineering University of California Berkeley California USA; ^2^ Civil, Environmental, and Geo‐Engineering University of Minnesota Minneapolis Minnesota USA; ^3^ St. Anthony Falls Laboratory University of Minnesota Minneapolis Minnesota USA; ^4^ Integrative Biology University of California Berkeley California USA; ^5^ Environmental Science, Policy, & Management University of California Berkeley California USA; ^6^ Civil, Environmental, and Mining Engineering University of Western Australia Perth Western Australia Australia; ^7^ Centre for Water and Spatial Science University of Western Australia Perth Western Australia Australia

**Keywords:** abscisic acid, drydown, isotope tracers, mutual information, rewatering, sap flow

## Abstract

Understanding and predicting plant water dynamics during and after water stress is increasingly important but challenging because the high‐dimensional nature of the soil–plant–atmosphere system makes it difficult to identify mechanisms and constrain behaviour. Datasets that capture hydrological, physiological and meteorological variation during changing water availability are relatively rare but offer a potentially valuable resource to constrain plant water dynamics. This study reports on a drydown and re‐wetting experiment of potted *Populus trichocarpa*, which intensively characterised plant water fluxes, water status and water sources. We synthesised the data qualitatively to assess the ability to better identify possible mechanisms and quantitatively, using information theory metrics, to measure the value of different measurements in constraining plant water fluxes and water status. Transpiration rates declined during the drydown and then showed a delayed and partial recovery following rewatering. After rewatering, plant water potentials also became decoupled from transpiration rates and the canopies experienced significant yellowing and leaf loss. Hormonal mechanisms were identified as a likely driver, demonstrating a mechanism with sustained impacts on plant water fluxes in the absence of xylem hydraulic damage. Quantitatively, the constraints offered by different measurements varied with the dynamic of interest, and temporally, with behaviour during recovery more difficult to constrain than during water stress. The study provides a uniquely diverse dataset offering insight into mechanisms of plant water stress response and approaches for studying these responses.

## Summary statement

The high‐dimensional nature of plant response and recovery to water‐limiting conditions creates scientific challenges. We examine how datasets that collect hydrological, physiological and meteorological variables simultaneously may help identify mechanisms and constrain predictions.

## 1 Introduction

The use of water by plants and the response of plants to variations in water availability play a central role in the terrestrial carbon, energy and water balance, as well as in determining plant health and ecosystem function (Bonan 2008). Plant water use and plant physiological responses to changing water status are complex interacting processes, which respond to exogenous climatic drivers, and are strongly mediated by edaphic (soil) conditions and phenotypic (physiological) plant traits. Identifying which of these climatic, edaphic and plant trait characteristics are responsible for observed plant status and water use phenomena is difficult (McLaughlin et al. 2017; Trugman et al. 2021). For example, Feng et al. (2018) interrogated a parsimonious model of the soil–plant–atmosphere continuum and found that nine non‐dimensional parameter groups were needed to describe plant water use or plant water potential in the model.

This ‘high dimensionality’ of plant–water interactions poses theoretical and modelling challenges. For example, many analyses of plant water dynamics attribute variations in plant water status or water use to a subset of physical or physiological conditions. In a high‐dimensional problem, however, such approaches risk omitted variable biases, which occur when an omitted variable impacts both the independent and dependent variables. Then, the effects caused by variations in the omitted variable can be incorrectly attributed to included independent variables (Butsic et al. 2017). For example, Feng et al. (2019) demonstrated that such misinterpretation could arise when inferring isohydry/anisohydry from measured plant water potentials. Understanding and predicting plant water use under water scarcity is generally high‐dimensional due to the complexity of describing the climatic, soil and plant trait drivers and their interactions (Feng et al. 2019; Martínez‐Vilalta and Garcia‐Forner 2017; Hochberg et al. 2018; Venturas et al. 2021; Powell et al. 2013; Trugman et al. 2018; Trugman 2022). Physical drivers including vapour pressure deficit (VPD; Grossiord et al. 2020; Novick et al. 2016), subsurface structure and processes (McLaughlin et al. 2020; McCormick et al. 2021; Dawson, Hahm, and Crutchfield‐Peters 2020; Hahm et al. 2019), and soil water potential (Novick et al. 2022), as well as plant functional traits (Greenwood et al. 2017; Skelton, West, and Dawson 2015; Anderegg et al. 2016; Wright et al. 2010) and outcomes such as hydraulic impairment due to xylem embolism (Choat et al. 2018) are all identified as key processes contributing to plant water use and status outcomes under dry conditions. Other physiological mechanisms including hormone‐induced stomatal closure (Tardieu and Davies 1993; Mittelheuser and Van Steveninck 1969; Raschke 1975; Davies et al. 1981; Nolan et al. 2017) and depletion of non‐structural carbohydrates (Sala, Woodruff, and Meinzer 2012; Sapes et al. 2021; Guo et al. 2020) and growth changes (Zweifel et al. 2021) may also be important. Generally, however, these drivers are studied independently, leaving many other features unmeasured or undercharacterized. Only rarely do studies simultaneously measure multiple physical and physiological variables. That is, most studies do not meet the demand of highly dimensional systems for holistic measurements to constrain and describe processes. This is particularly challenging for modelling, when a need to identify many parameters with scarce data produces ‘equifinality’—a condition whereby multiple model parameterisations or formulations produce equivalently good model performance (Beven 2006). Poorly constrained model parameters, in turn, lead to more uncertain model predictions.

Challenges around high dimensionality are common in environmental systems, and other disciplines can offer case studies and strategies for tackling high‐dimensional systems. Catchment hydrology, for instance, has demonstrated the value in collecting and combining measurements of state variables (e.g., soil moisture), fluxes (e.g., streamflow) and source information (e.g., tracers). The Maimai catchments in New Zealand have been intensively studied using this diversity of approaches, enabling a significant improvement in understanding the mechanisms and dynamics of water flow in the catchment (McGlynn, McDonnel, and Brammer 2002; McDonnell et al. 2021). Similar benefits could be expected from collecting concurrent state, flux and source data within the soil–plant–atmosphere system.

Here we collect a suite of diverse measurements of physiological, meteorological and hydrological variables during a drydown and re‐wetting experiment, thereby characterising the plant water fluxes, water status and water sources. We share this detailed data in the hope that it can be used for model development and testing of this high‐dimensional system. We focus our own analysis of the data on examining its benefits for advancing (i) mechanistic interpretation of the plant–soil–atmosphere system and (ii) predictive constraints on models of the system. In the first case, we synthesise our multiple measurements to identify mechanisms and drivers of plant water stress response and recovery, identifying situations where bringing in additional variables can advance interpretation. In the second case, we draw on information theory to quantify the value and limitations of the different measurements for constraining plant water dynamics. The data collection through this experiment creates an opportunity to explore not just the mechanisms that lead to observed plant water use behaviour, but also provides an opportunity to examine how the data collected about this high‐dimensional system can be used to reach conclusions about the system. Together, the data and analyses advance understanding of plant water stress response and recovery, while providing practical insights into the measurement and analysis of such processes.

## 2 Methods

A number of advances from across different disciplines have enabled better characterisation of different aspects of plant–water interactions, which we leveraged for this experiment. While the underlying principles of many of these measurements are not new, technical advances have made measurements easier, faster and cheaper. Providing important information about the gradients driving water fluxes, advances in sensors and instruments have made measurements of water potentials in soils and plants more accessible (Novick et al. 2022). Characterising xylem vulnerability to embolism, a physiological trait thought to play a key role in plant drought response and mortality, has likewise become easier in recent years (Brodribb et al. 2016, 2017). Sensors for measuring water flow through plants (sap flux) have also improved, allowing for well‐resolved measurements of water use fluctuations across species and environments (Poyatos et al. 2021; Forster 2020). For ascertaining below‐ground dynamics, stable isotopes have proved a useful tool for identifying plant water sources (Dawson and Ehleringer 1991; Ehleringer and Dawson 1992; Dawson 1996; Nehemy et al. 2021). Water molecules contain stable isotopes of both hydrogen (^1^H and ^2^H or D) and oxygen (^16^O, ^18^O, and the far less abundant ^17^O). The abundance of these stable isotopes in a given sample of water reflects the mixing and fractionation (including evaporation and condensation) processes the sample has experienced. Root water uptake represents a generally non‐fractionating process, such that the isotopic composition of water in the plant is representative of the mixture of sources from which the plant drew water (Dawson and Pate 1996; Flanagan, Ehleringer, and Marshall 1992; Bariac, Jusserand, and Mariotti 1990; Wershaw et al. 1966; Dawson and Ehleringer 1993; Thorburn, Walker, and Brunel 1993; Walker and Richardson 1991; Washburn and Smith 1934; White et al. 1985; Ziegler et al. 1976; although fractionation has been shown to occur in some halophytic [Lin and Sternberg 1993] and xerophytic [Ellsworth and Williams 2007] species). Measurement of stable isotopes of water in plants and potential plant water sources can thus enable inference about which of these water sources are used by plants and in what ratio. Of course, this requires that distinct water sources are isotopically distinguishable. While natural abundance water isotopes are still able to provide insight in many settings, the addition of isotopically enriched water tracers can provide greater identifiability of water sources (Beyer et al. 2016).

### 2.1 Experimental Setup and Timeline

**Figure 1:**
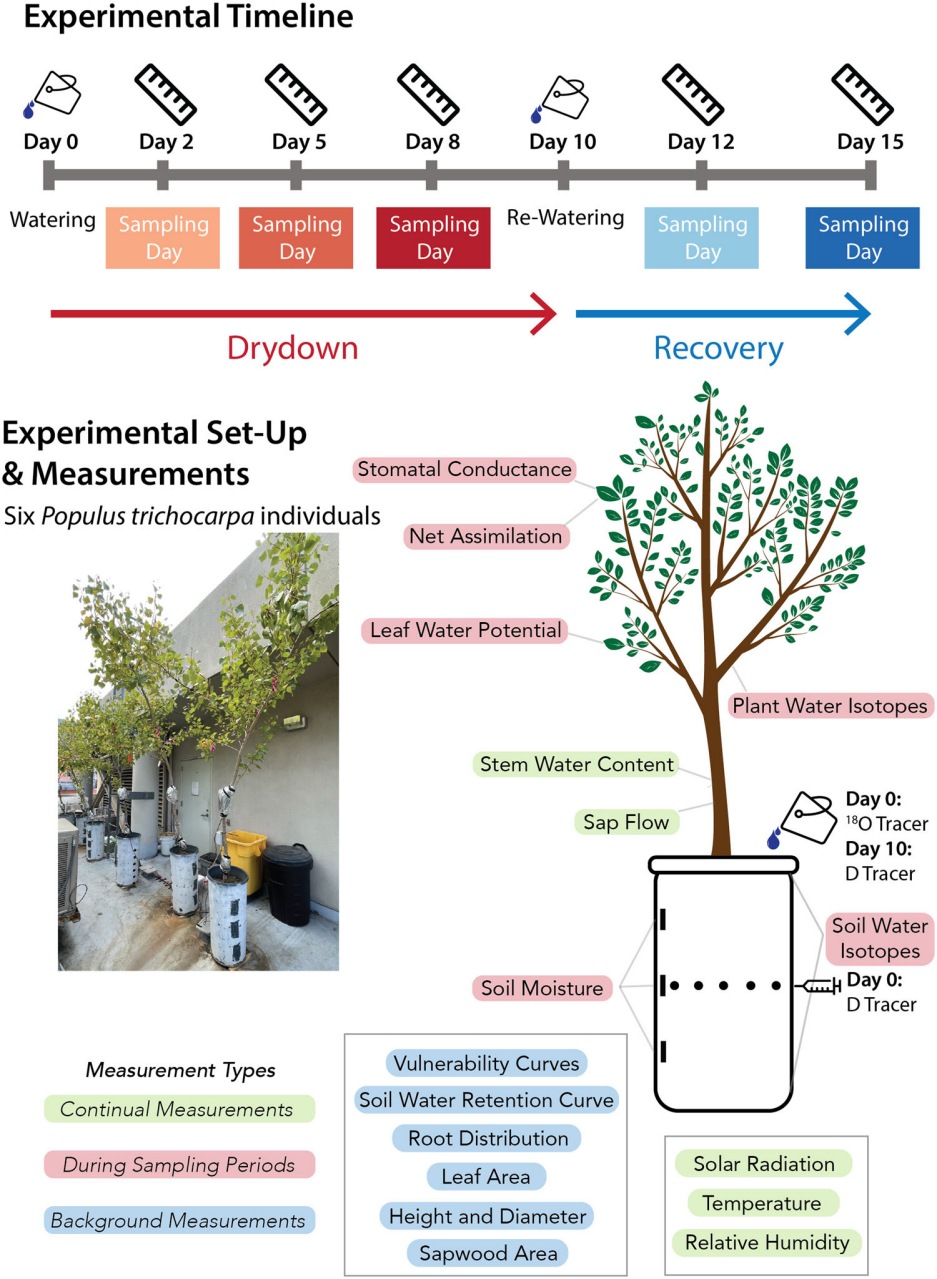
The experimental setup consisted of six potted *Populus trichocarpa* trees on which various measurements were made to characterise plant function, environmental conditions and fluxes of water and carbon. Some of these measurements were continual (green), some were done only during select intensive sampling days (red) and others acted as background measurements that were completed before the start or following the conclusion of the experiment (blue). On Day 0, an ^18^O tracer was applied to the soil surface and a D tracer was injected halfway down the ≈ 65 cm soil column. Three intensive sampling days occurred on Days 2, 5 and 8, during the drydown period. The trees were then rewatered on Day 10 and a D tracer was applied to the surface. During the subsequent recovery period, two intensive sampling days occurred on Days 12 and 15. In this and the subsequent result figures, the drydown sampling days are represented with progressively darker shades of red, and the recovery sampling days are represented with progressively darker shades of blue.

The experiment was conducted on six potted *Populus trichocarpa* individuals (Figure 1). The trees were transplanted as saplings into ≈65 cm tall, ≈40 cm diameter pots ($\approx 0.08\;{\;m\;}^{3}$ soil volume) nearly 2 years prior. The trees were grown outdoors under ambient conditions in Berkeley, California, with frequent watering to ensure well‐watered conditions during sapling growth. During the experiment, the trees were subjected to a 10‐day drydown followed by a rewatering and 6‐day recovery period, as follows.

On Day 0 (6 July, 2021), two water sources with different isotopic labelling were used to water each plant. At the soil surface, 5.5 L of an ^18^O enriched water tracer (≈100‰δ ^18^O prepared from ICON ${\;H\;}_{2}$ ^18^O ≥ 98 atom%) was applied to each plant. Later that day (≥ 3 h), 8.3 L of a D‐enriched water tracer (≈350‰δD prepared from Sigma‐Aldrich ${\;D}_{2}O\;$ 99.9 atom%) was injected into the centre of the soil column through ports located 28–30 cm below the surface. Isotopic compositions of the individual tracers applied to each tree are reported in Table S1.

Following initial watering/tracer application, the trees were subjected to a 10‐day drydown period with no other water applied. On Day 10 between 9:30 and 10:00, the trees were rewatered with 15 L of unlabelled tap water (−12.2‰δ ^18^O, −88.0‰δD) applied at the surface, to ensure thorough soil re‐wetting. Later the same day between 12:30 and 13:30, 4 L of D‐labelled water (≈350‰δD prepared from Sigma‐Aldrich ${\;D}_{2}O\;$ 99.9 atom%) were applied to the surface. The experiment continued for another 5 days, concluding 15 days after initial watering. Tracer application was informed by a pilot test, which is described in Section S2.

During the experiment, background, scheduled and continual measurements were made to characterise plant traits, plant function and environmental variables, as summarised in Figure 1. Background measurements were conducted on soil and well‐watered plant samples independently of the experiment, as well as on a subset of trees that were destructively sampled at the end of the experiment. Scheduled measurements on select sampling days were undertaken on Days 2, 5, 8, 12 and 15. Continual measurements were made with in situ sensors for the duration of the experiment.

### 2.2 Background Measurements

Background measurements were made before and after the experiment to characterize the trees and soil. Three additional *P. trichocarpa* were grown under identical conditions to the experimental trees, and samples of their leaves and stems were used to measure the vulnerability of stem and leaf xylem to embolism. The $P_{50}$ values were −2.53 and −1.48 MPa for the leaves and stems, respectively, indicating reverse vulnerability segmentation. Through modelling experiments in Wilkening et al. (2023), we find that this pattern of segmentation could support the prioritisation of protecting the whole‐plant hydraulic pathway, as compared to the stem hydraulic function with conventional vulnerability segmentation with more vulnerable leaves. The optical method was used to make these measurements, and further methodological details and results are reported in Wilkening et al. (2023). Gas exchange response curves for light and ${\;\text{CO}\;}_{2}$ ($A∕C_{i}$) were also measured for a subset of trees before the start of the experiment. Further details and results are presented in Section S3.

Following the experiment, the height of each experimental tree and its stem diameter at the sap flow installation site was measured. Three of the trees (Trees 1, 4 and 5) were cut at the base at soil level. Sapwood area was calculated at the sap flow sensor installation site from the stem cross‐section based on the measured sapwood diameter. Morphological data are reported in Section S4.

At the conclusion, soil samples were removed at 10‐cm intervals from the surface to 60‐cm depth. The sections (approximately 2.5 cm tall, 2.5 cm wide and 11 cm long as measured into the pot) were cut away from the remaining soil, sealed in plastic bags and frozen until processing. During processing, each soil sample was thawed, soaked in water and sieved to separate root sections from soil. Washed root samples were oven‐dried at $60^{\circ }$C for 1 week and weighed to provide an estimate of the root mass profile with depth. Root mass profiles are shown in Section S5.

Two surface soil samples were taken from the remaining trees and used to characterize the soil water retention curve using the METER HYPROP 2 and WP4C instruments (Schelle et al. 2013; Devices 2010; Scanlon and Andraski 2002; Bezerra‐Coelho et al. 2018). A bimodal van Genuchten model was fit to the measurements using the SoilHyP package in R (Durner 1994; Van Genuchten 1980; Dettmann 2022; R Core Team 2022). Results and further details are reported in Section S6.

### 2.3 Continual Measurements

A number of continual measurements were made throughout the experiment at daily or sub‐daily frequencies. In situ sensors were used to record measurements of meteorological conditions, sap flow and stem water content at 15‐min intervals. Samples of transpired water were collected daily, and leaf‐fall from the crowns was collected daily to measure leaf loss. Crown conditions were also monitored using 10‐min interval time‐lapse photography during daylight hours.

Meteorological conditions were measured using a temperature and relative humidity probe (Campbell Scientific HMP50) and pyranometer (Campbell Scientific LI200RX) installed at crown height (2.3 m above ground) adjacent to the trees. Data were recorded using a Campbell Scientific CR1000 every 15 min. The temperature and relative humidity were then used to compute the atmospheric VPD at each time point (Murray 1967).

Sap flow sensors (Implexx) installed on each tree logged measurements (Campbell Scientific CR1000) every 15 min. The sensors measured heat velocity via the Dual Method Approach (DMA), which combines the Heat Ratio Method and Tmax heat pulse approaches (Forster 2020). The sensors also measured stem water content. Sap flow measurements were cleaned by removing time points where heat velocity values showed large negative excursions (heat velocity 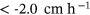), which indicated sensor malfunction (≈ 0.4% of measurements). Heat velocity measurements (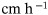) were converted to sap flux (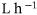) using the sapwood area measurements previously described. Finally, a Hampel Filter was used to identify and replace outliers with the rolling median based on the sap flow values (Poyatos et al. 2021) (≈ 14% of measurements). The baseline for sap flow was then corrected based on the minimum measured value, assumed to represent time points without active transpiration. Wound diameter around the probe installation sites was also measured and recorded for each stem.

To monitor tracer breakthrough, transpired water was collected daily from a portion of a sunlit branch on each tree, using different branches on subsequent days for each tree. A clear plastic bag was securely taped around the branch in the morning and left on the tree for at least 6 h. Condensed transpiration was collected into vials, sealed and frozen until isotopic analysis. Results and further analytical details for these measurements are presented in Figure S8.

A time‐lapse camera (Brinno TLC200) was installed, which recorded images of the tree crowns at 10‐min intervals during daylight hours. Throughout the experiment, any leaves lost by the trees, either through removal for other samples or natural abscission, were collected and their areas were measured using a leaf area meter (LICOR LI‐3100C). Where possible, the tree from which the leaf originated was recorded. At the end, all remaining leaves on Trees 1, 4 and 5 were removed, scanned and their total area recorded.

### 2.4 Sampling Period Measurements

On designated intensive sampling days, leaf water potential, leaf gas exchange and soil water content were measured, and soil and plant tissue samples were taken for water isotope analysis.

Predawn (05:00–05:45) and midday (13:30–14:30) leaf water potential measurements were made for each tree, with at least two replicates at each time point for each tree. Leaves were excised and immediately placed into a pressure chamber (PMS Instruments Model 1505D) to measure water potential (Boyer 1967; Scholander et al. 1965).

Leaf gas exchange measurements of ${\;\text{CO}\;}_{2}$ assimilation and stomatal conductance were also made in the morning (10:15–11:15) and in the afternoon (14:30–15:30) on Days 2, 5 and 12 using a portable photosynthesis system (LI‐COR LI‐6800) (Field, Ball, and Berry 1989; Von Caemmerer and Farquhar 1981). At least two replicates were made for each tree and time point, on the same branch for each tree for every set of measurements to help control for differences in shading before measurement between the sampling days. Measurements were made under constant chamber conditions (${\;\text{CO}\;}_{2}$ at 425 ppm, 70% relative humidity, $1500\;\mu {\;\text{mol m}\;}^{-2}\;{\;s\;}^{-1}$ light source and temperature set to leaf reference). Upon enclosing the leaf in the chamber, measurements were recorded once gas exchange measurements had stabilised according to instrument stability criteria or once 3 min had passed, whichever came first.

Soil water content ($V_{water}∕V_{soil}$, %) was measured 10, 30 and 50 cm below the surface using a handheld probe (Campbell Scientific HS2 with CD659 probe) inserted through side ports in the containers. Between measurements, ports were sealed to prevent evaporation.

Soil samples for water isotope analysis were collected at 10‐cm intervals between 0 and 60 cm from the surface. For each sample, soil was collected by drilling a new hole through the side of the containers from which soil was then removed and the hole was resealed to prevent water loss. Soil samples were sealed in vials and immediately frozen until ready for extraction and analysis.

In the late afternoon (14:30–16:30), plant tissue samples for water isotope analysis were collected from each tree. Samples of mature woody stems were collected, sealed in vials and immediately frozen for storage.

### 2.5 Isotope Sample Processing and Analysis

All isotope sample processing and analysis was done at the UC Berkeley Center for Stable Isotope Biogeochemistry. Water from plant and soil samples was extracted using cryogenic vacuum distillation. Water was extracted from the samples under vacuum at $100^{\circ }$C for at least 1 h (West, Patrickson, and Ehleringer 2006). All transpired water samples were analysed using Isotope Ratio Mass Spectrometry (IRMS) (Thermo Delta V Plus). Isotopic abundances of hydrogen and oxygen are expressed in delta notation:

(1) $$\delta =\left(\frac{R_{sample}}{R_{standard}}-1\right)\times 1000$$

where R is the ratio of the heavy to light isotope (D/H and ^18^O/^16^O). The standard is Vienna Standard Mean Ocean Water (VSMOW). All extracted stem water and soil water samples were analysed using Isotope Ratio Infrared Spectrometry (IRIS) (Los Gatos Research). A representative subset of the stem and soil samples was also analysed using IRMS to corroborate the values measured using IRIS. These methodological comparison data are reported in Section S8. Long‐term precision for IRIS measurements was 1.3‰ and 0.52‰ for δD and $\delta^{18}$O, respectively. Long‐term precision for IRMS measurements was 0.81‰ and 0.071‰ for δD and $\delta^{18}$O, respectively.

### 2.6 Mutual Information of Measurements

To provide a quantitative assessment of the ‘value’ of different measurements, we use concepts and metrics from information theory. Information theory offers valuable techniques for assessing the relationships between variables in complex systems (Goodwell et al. 2020) and has been shown to be valuable for applying to ecophysiological problems (Bassiouni and Vico 2021). In particular, we use mutual information:

(2) I(X;Y)=H(X)−H(X∣Y)

where for two variables X and Y, the mutual information (I(X;Y)) is the difference between the marginal entropy of X (H(X)) and the conditional entropy (H(X∣Y)). As such, mutual information describes how knowledge of one variable can reduce uncertainty in another variable (Ross 2014). Higher mutual information values indicate a greater reduction in uncertainty and a stronger statistical relationship between the variables. Lower values of mutual information indicate that variables are more independent of one another, such that one does not constrain the other. Mutual information is non‐parametric, enabling assessment of the often non‐linear dynamics in soil–plant–atmosphere systems that is also independent of any assumed relationships between different variables that might emerge from our current theories. Variables that are more correlated, in a general sense, will have greater mutual information values, but unlike a correlation coefficient, mutual information can quantify non‐linear relationships. Here, we calculate the mutual information between different measurements and other measurements that represent typical targets of predictive models. This includes plant water fluxes (represented by mean daily sap flow) and plant water status (represented by predawn and midday leaf water potentials). The measurements were summarised to daily values, such that variables are compared at a common measurement frequency. Full details of each measurement are presented in Table 1. The summary calculations using the isotopic measurements to assess plant water sources are further detailed in Section S9. Mutual information values are calculated for the full experiment, as well as separately for the drydown and recovery periods.

**Table 1:** The experimental data are summarised for different variables representing different aspects of the soil–plant–atmosphere system.

Measurement	Calculation
Physical environment variables	
Soil moisture	Mean across depths
VPD	Daily mean
Solar irradiation	Daily mean
Plant water status	
Predawn leaf water potential	Daily mean
Midday leaf water potential	Daily mean
Cumulative leaf loss	Cumulative to date
Plant fluxes	
Daily mean sap flow	Mean during daylight hours
Maximum stem water content	Daily 95th percentile
Minimum stem water content	Daily 5th percentile
${\;\text{CO}\;}_{2}$ assimilation	Daily mean
Stomatal conductance	Daily mean
Plant water sources	
Mean water uptake depth	Calculated from soil and plant isotopes (see Section S9)

*Note:* These summarized variables are then used for the mutual information calculation

## 3 Results

Results are presented in four categories, corresponding to the processes and dynamics they represent. Meteorological and soil moisture data are presented in Section 3.1. Sap flow and stem water content data are presented in Section 3.2. Leaf water potentials and gas exchange measurements are presented in Section 3.4. Isotopic measurements are presented in Section 3.3. Mutual information values are presented in Section 3.5. Additional data are reported in the Supplementary Information.

### 3.1 Physical Environmental Measurements

Full meteorological data over the experimental period are presented in Figure S11. Overall, the diurnal cycles of air temperature, VPD and solar irradiance were relatively consistent over the study period. Daily high temperatures ranged from approximately 18°C to 25°C and low temperatures ranged from approximately 12°C to 15°C. Daily maximum VPD ranged from approximately 0.75 to 1.75 kPa and overnight minimums ranged from approximately 0.2 to 0.3 kPa. Days 2–4 experienced relatively higher daytime temperatures and VPD values than the rest of the experimental period. Daily patterns of solar irradiance were quite consistent, with daily maximum values near $1000\;{\;\text{W m}\;}^{-2}$.

**Figure 2:**
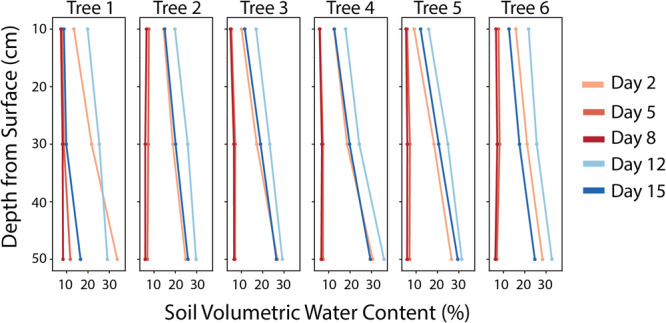
The soil moisture at different depths across the sampling days is shown for each tree. Measurements of soil volumetric water content ($V_{water}∕V_{soil}$, %) were made at depths of 10, 30 and 50 cm below the surface. The different sampling days are denoted with different line/point colours, with the drydown period in shades of red and the recovery period in shades of blue. [Color figure can be viewed at wileyonlinelibrary.com]

Soil moisture depth profiles on sampling days for each tree are shown in Figure 2. The corresponding soil water potential values are in Section S11, which were converted to water potential values using the water retention measurements and curve fits presented in Section S6. On Day 2, water content values ranged from approximately 10% to 30%, with lower values near the surface and higher values at depth. By Day 5, there were declines in soil moisture across all depths and trees. Values ranged from approximately 5% to 10%, with more uniform profiles across depths. Between Days 5 and 8, there were minimal changes in soil moisture across trees and depths. Following rewatering on Day 12, soil moisture values ranged from approximately 15% to 35%. For most trees, soil moisture on Day 12 was generally higher than on Day 2, but showed similar profiles with lower values near the surface and higher values at depth. By Day 15, there were some declines in soil moisture, although the differences were less than that observed between Days 2–5, with Day 15 soil moisture values ranging from approximately 10% to 30% for most trees. Tree 1 was an exception, showing greater declines to a more uniform soil moisture distribution.

### 3.2 Plant Water Flux Measurements

**Figure 3:**
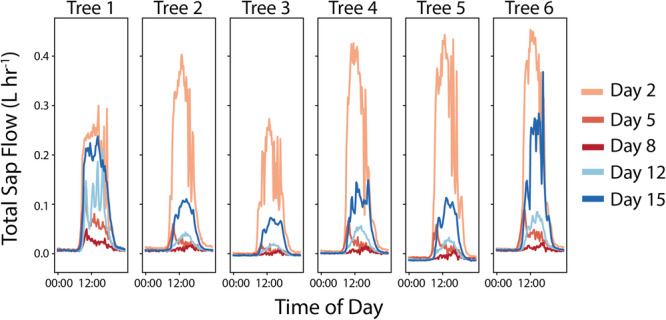
The diurnal cycles of total sap flow (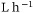) across the different sampling days are shown for each of the six trees across the panels. The different sampling days are denoted with different line colours, with the drydown period in shades of red and the recovery period in shades of blue. [Color figure can be viewed at wileyonlinelibrary.com]

**Figure 4:**
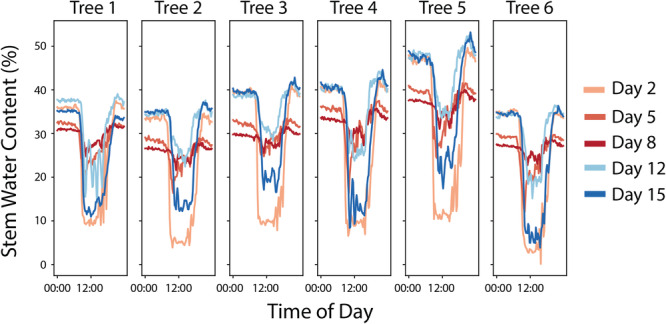
The diurnal cycles of stem water content (%) across the different sampling days are shown for each of the six trees across the panels. The different sampling days are denoted with different line colours, with the drydown period in shades of red and the recovery period in shades of blue. [Color figure can be viewed at wileyonlinelibrary.com]

Changes in sap flow and stem water content in each tree are compared across each of the intensive sampling days in Figures 3 and 4, respectively. Full time series of both variables for the duration of the experiment are presented in Section S12. While Day 2 was the first full measurement day, the sap flow rates are comparable to those after watering on Day 0. The trees experienced a pronounced decline in sap flow between Days 2 and 5, and by Day 8 most trees had negligible sap flow. Following rewatering on Day 12, most trees showed only a minimal increase in sap flow. On Day 15, sap flow rates further recovered, but most trees still remained below the rates observed on Day 2. Tree 1 shows a somewhat different pattern than the other trees, with relatively higher rates of sap flow on Days 5 and 8. During the recovery period, Tree 1 showed greater recovery of sap flow, with rates on Day 15 returning close to the initial values on Day 2. For the stem water content, there was a dampening of the diurnal cycle for all the trees over the course of the drydown, with less drawdown during the day and lower water content recovery overnight. Following rewatering, the overnight stem water content values on Day 12 returned to similar values as Day 2, although there was less daytime drawdown of stem water content. By Day 15, the cycles for Trees 1, 4 and 6 had returned close to what they were on Day 2, but the remaining trees still showed less daytime drawdown of water.

**Figure 5:**
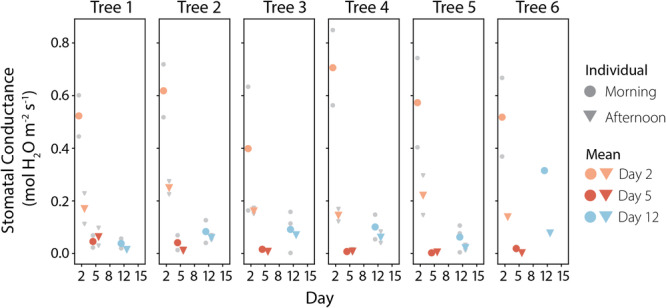
Stomatal conductance measurements (mol ${\;H}_{2}O\;\;{\;m\;}^{-2}\;{\;s\;}^{-1}$) from the sampling days for each tree are shown. Individual measurements are shown as grey points, with the morning measurements (10:15–11:15) shown as circles and the afternoon measurements (14:30–15:30) shown as triangles. The means for each day and sampling time are shown with coloured points. [Color figure can be viewed at wileyonlinelibrary.com]

Gas exchange measurements of stomatal conductance are presented in Figure 5, and the corresponding measurements of ${\;\text{CO}\;}_{2}$ assimilation, which showed similar patterns, are shown in Figure S15. Stomatal conductances and assimilation rates for each tree were generally highest on Day 2. Most trees showed some decline in both stomatal conductance and assimilation rate in the afternoon measurements on Day 2, relative to the morning measurements from that day. On Day 5, most trees had stomatal conductances and assimilation rates close to zero in both the morning and afternoon. The exception was Tree 1, which showed values that were higher relative to the other trees on that day, but were still much lower than what had been measured for Tree 1 on Day 2. On Day 12, most trees showed an increase in stomatal conductance and assimilation rate, particularly Tree 6 which returned almost to the values observed on Day 2. An exception was Tree 1, which had assimilation rates and stomatal conductance values that were lower than those on Day 5. Additional figures showing the coordination of changes in stomatal conductance and assimilation rates are shown in Section S14.

### 3.3 Plant Water Sources

**Figure 6:**
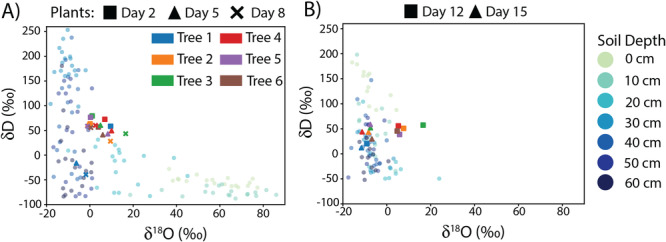
The soil and stem water isotope samples during the drydown period (A) and recovery period (B) are plotted in dual isotope space. The soil water isotopes are plotted as circles, with darker shades corresponding to lower depths. For the stem water isotopes, the sampling days are represented with different symbol shapes. The symbol colour corresponds to the tree. [Color figure can be viewed at wileyonlinelibrary.com]

The isotopic composition of stem and soil waters are shown for the drydown and recovery periods in Figures 6A,B, respectively. The depth profiles of δD and $\delta^{18}$O in the soil water samples are presented in Section S15. During the drydown period, the uppermost soil layers were relatively enriched in ^18^O and depleted in D, the mid‐depth soils were enriched in D and depleted in ^18^O, and the deepest soil layers were depleted in both isotopes. Most of the plant waters fell at intermediate values of both δD and $\delta^{18}$O ($\delta \;D\approx 25–75‰,\delta^{18}O\approx 0–\;20‰$). The stem waters for Tree 1 on Days 5 and 8 show excursions towards less enriched values for both isotopes. During the recovery period, the uppermost soil layers were enriched in D, and all soil layers were relatively (as compared to the drydown period) depleted in ^18^O. On Day 12, most of the stem waters have intermediate δD values close to 50‰ but have $\delta^{18}$O that are more enriched (0–20‰) than most of the soil waters. The stem water for Tree 1 had slightly less enriched δD and did not show the same enrichment in $\delta^{18}$O relative to the soil water. On Day 15, all the stem waters have similar intermediate values of δD, but show a shift towards more depleted values of $\delta^{18}$O that are within the range of those in the soil.

### 3.4 Plant Water Status

**Figure 7:**
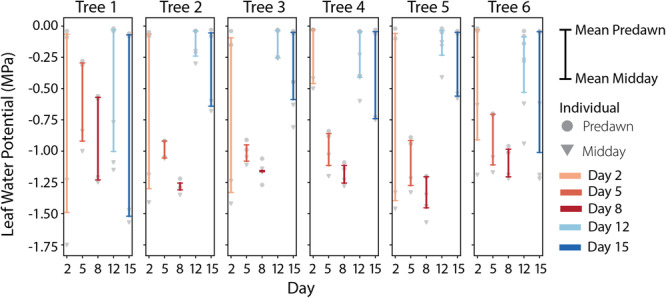
Leaf water potentials (MPa) from the sampling days for each tree are shown across the panels. Individual measurements are shown as grey points, with the predawn measurements (5:00–5:45) as circles and the midday measurements (13:30–14:30) as triangles. For each day, the range of the mean predawn and mean midday leaf water potential is shown with a coloured bar, where the upper endpoint corresponds to the mean predawn leaf water potential and the lower endpoint corresponds to the mean midday leaf water potential. [Color figure can be viewed at wileyonlinelibrary.com]

The leaf water potential measurements for each tree over the course of the experiment are shown in Figure 7. On Day 2, the predawn water potentials for all the trees were close to 0 MPa and the mean midday water potentials ranged from approximately −0.5 to −1.5 MPa. As the drydown progressed, the predawn water potentials became more negative. Between Days 5 and 8, the mean midday water potentials became more negative and the difference between the predawn and midday water potentials became smaller. However, for some of the trees, the midday water potentials on Days 5 and 8 were still less negative than those on Day 2. On Day 12, the predawn leaf water potentials returned close to 0 MPa for all trees, but the midday leaf water potentials were less negative than they were on Day 2. On Day 15, the predawn water potentials for all trees remained close to 0 MPa, but the midday water potentials became more negative, with Trees 1, 4 and 6 having midday leaf water potentials equal to or less than those on Day 2.

**Figure 8:**
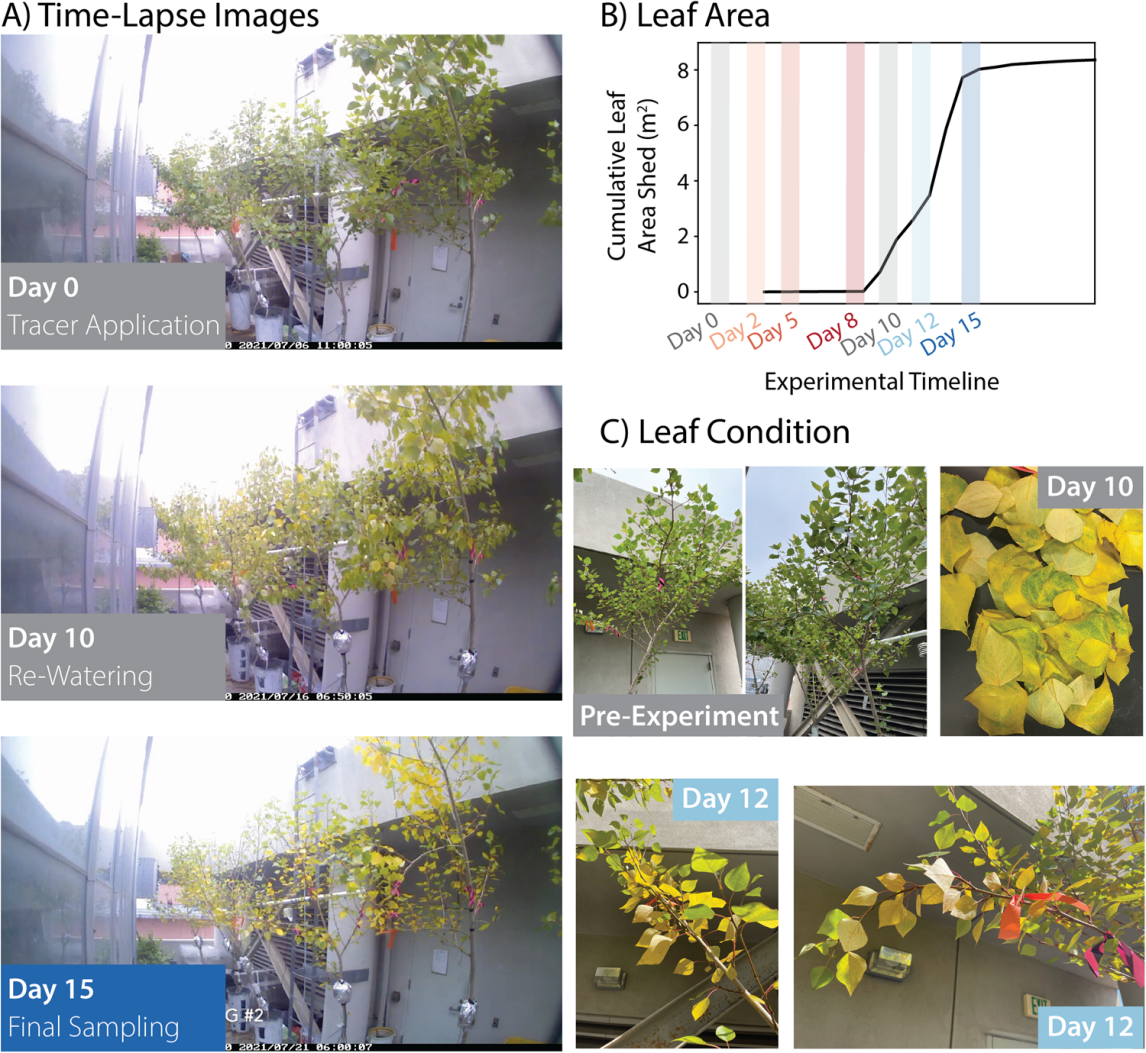
The changes in leaf condition and leaf area were recorded over the course of the experiment through images and through measurements of leaf area of dropped leaves. Time‐lapse images (A) show the progression of leaf yellowing and loss over the drydown (Days 0–10) and recovery periods (Days 10–15). The cumulative area of shed leaves over the course of the experimental period is shown in B, with the sampling (coloured) and watering (grey) days denoted with highlighted bars. This data represents the collective leaf area shed and collected from all trees. Close‐up images (C) further show changes in leaf condition in individual leaves and branches and the appearance of shed leaves that were collected. [Color figure can be viewed at wileyonlinelibrary.com]

Figure 8 shows images and measurements of changes in crown condition and leaf shedding over the course of the experiment. At the start of the experiment, the trees were fully leafed out with green leaves. By the end of the drydown period, all the trees had begun to exhibit some leaf yellowing and shedding. The yellowing continued to progress during the initial days following rewatering. The trees also continued to shed leaves, with the majority of leaf shedding occurring between Day 9 and Day 15. By Day 15, the canopies were notably sparser. Of the remaining leaves, greener leaves were typically at the distal end of branches, with leaves closer to the trunk showing more yellowing. From a purely visual assessment, we estimate leaf area loss on a per‐tree basis was on the order of 30–60%; however, there was still substantial yellowing through the crown in the remaining leaves at the end of the experiment.

### 3.5 Mutual Information

**Figure 9:**
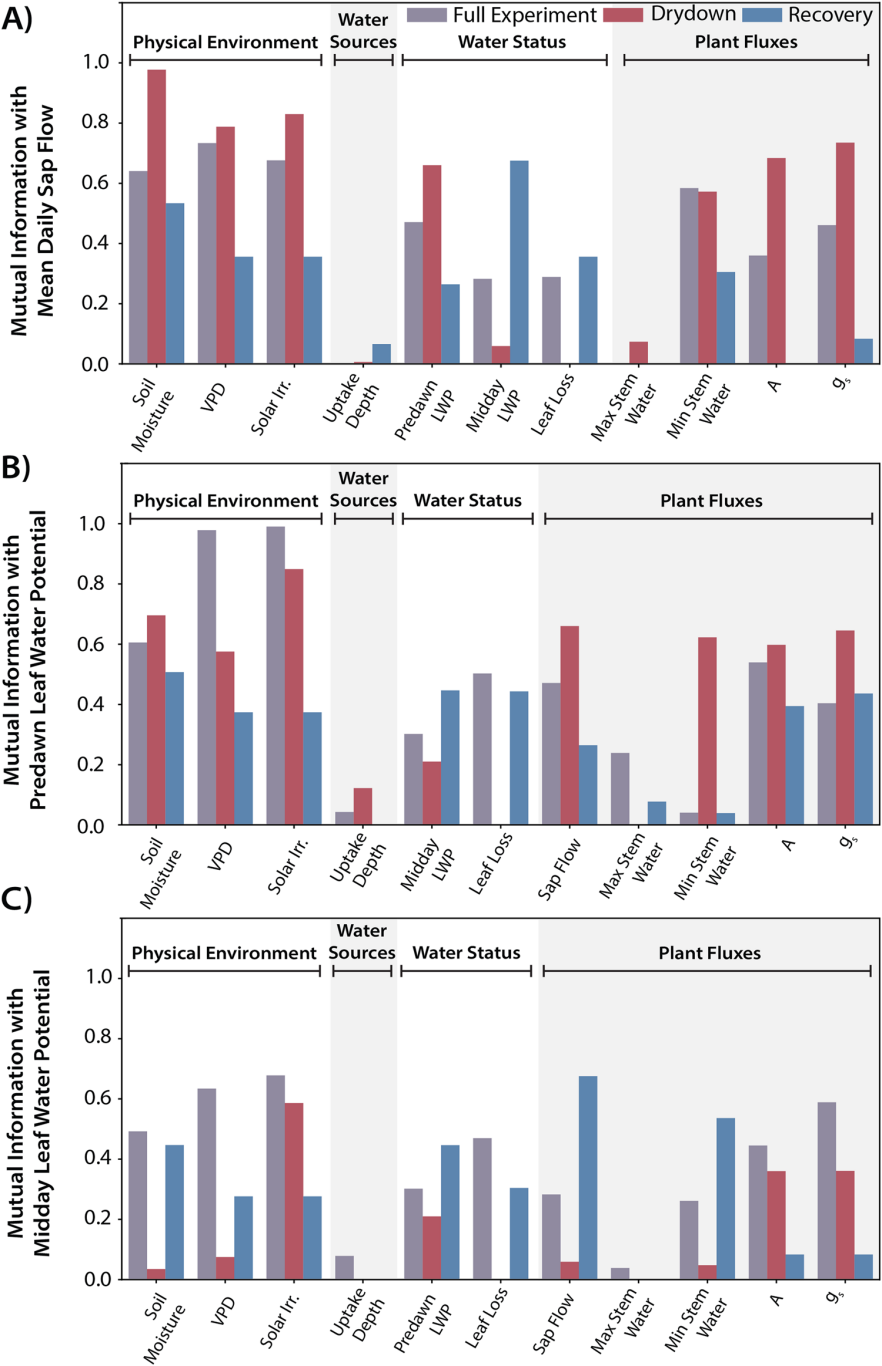
Mutual information values between various measurements (x‐axis) and the mean daily sap flow (A), predawn leaf water potential (B) and midday leaf water potential (C) are shown with different coloured bars for the different time periods, including the full experimental period (purple), the drydown period (red) and the recovery period (blue). Shading separates the groupings of measurements describing (L to R) the physical environment, plant water sources, plant water status and plant fluxes. Higher mutual information values indicate a greater reduction in uncertainty for the target variable with the information from that measurement. [Color figure can be viewed at wileyonlinelibrary.com]

The mutual information values are shown in Figure 9 for sap flow (Figure 9A), predawn leaf water potential (Figure 9B), and midday leaf water potential (Figure 9C). Additional pairwise mutual information values are reported in Section S16. The sap flow mutual information values varied across the different measurements, as well as between the different time periods within the experiment. For the full experimental period, VPD had the highest mutual information with sap flow with a value of 0.73. The other measurements had values ranging from approximately 0.28 to 0.68, with the exception of the mean water uptake depth and maximum stem water content which had mutual information values of 0.0 with sap flow. Sap flow mutual information values were generally higher for the drydown period than during the recovery. During the drydown period, mean soil moisture had the highest value (0.98), although VPD and solar irradiation also had values greater than 0.75. During the recovery period, midday leaf water potential and soil moisture had the highest mutual information values with sap flow, 0.68 and 0.53, respectively.

The predawn and midday mutual information values similarly varied across the measurements and time periods. For the mutual information with predawn water potential during the full experimental period, the physical environment variables had the highest mutual information ranging from 0.61 to 0.99. The physical environmental variables also had relatively high mutual information during the drydown period (ranging 0.58–0.85), but the plant flux variables, with the exception of the maximum stem water content, also showed higher values (ranging 0.60–0.66). Mutual information values with predawn water potential were also generally lower for most variables during the recovery period. For midday leaf water potential mutual information, values were generally lower across all periods and measurements than compared to those for sap flow and predawn water potential, with all mutual information values less than 0.70. For the full experimental period, the physical environmental variables and gas exchange measurements had the greatest mutual information with midday leaf water potential (0.49–0.68). During the recovery period, the mean daily sap flow, minimum stem water content, soil moisture and predawn leaf water potential also had values in a similar range (0.45–0.68). For both predawn and midday leaf water potential, the mutual information with mean water uptake depth and maximum stem water content was below 0.25 for all time periods, and equal to 0.0 for a number of those cases.

## 4 Discussion and Conclusions

Taken together, the data help to reveal some of the patterns in responses and interactions that occurred over the drydown and recovery periods, as well as what drivers could be behind these patterns. Over the course of the experiment, the meteorological conditions were relatively consistent. As such, we can consider water availability to be the primary environmental driver behind the observed variations. In general, the drydown period showed declines in plant water potentials, sap flow rates, maximum daily stem water content and diurnal stem water content fluctuations. There were declines in assimilation rates in concert with stomatal closure. The plant water isotopes showed relatively consistent values that were intermediate relative to the soil, indicating the use of water from a mixture of depths throughout the drydown period. During the recovery period, plant water potentials and daily maximum stem water content were rapidly recovered. However, there was a delayed and, for many trees, only partial recovery of sap flow rates, assimilation rates and stomatal conductance. In this way, these trees seemingly exhibited both low resistance and low resilience to water stress. The plant water isotopes initially indicated a water source more enriched in $\delta^{18}$O than any of the soil water sources followed by a shift towards water uptake that occurred over some mixture of soil depths. The trees also experienced significant yellowing and leaf drop that occurred largely during the recovery period.

We do note that the drydown occurred fairly rapidly. However, these were sizeable trees with roots throughout their finite containers, such that the soil moisture was depleted quickly. Unlike some other drydown experiments (Blackman et al. 2019), we also did not pre‐expose the trees to any water limitations before the drydown. Silim et al. (2009) previously showed that poplars pre‐conditioned to water limitations exhibited more conservative $g_{s}$ values and water use in subsequent drought treatments than compared to individuals that were not pre‐conditioned. As such, the lack of pre‐treatment in our study could have also contributed to the more rapid depletion of soil moisture with higher initial water fluxes. While the speed of the drydown in our potted trees means that it does not necessarily act as a direct analogue for natural settings, it does not impede the utility of this dataset for assessing the utility of measurements for interpreting and constraining system behaviour.

Synthesis of these measurements can reveal the dynamics and timelines of plant water stress response and recovery during the experiment and potential driving mechanisms within the plant. We first explore this qualitatively, by examining how variables, either in isolation or in combination, can be used to test hypotheses for mechanisms behind the observed dynamics. We then use the quantitative assessments from the mutual information analysis to discuss how the level of constraint offered varies by measurement and over time. Finally, we discuss how, more broadly, these data can also be used to analyse the challenges and opportunities for better constraining studies of soil–plant–atmosphere systems.

### 4.1 Dynamics and Mechanisms of Plant Water Stress Response and Recovery During the Experiment

During the drydown, the trees showed notable declines in function and water status. Following the rewatering, most of the measures of plant function and water status showed recovery, but this recovery notably varied in timing and magnitude across the different measures. There are a number of potential explanations for this observed behaviour which can be assessed through synthesis of the various data collected. Given the delayed and partial recovery of transpiration rates, it indicates that the imposed water limitation was likely more severe than just mild water stress for the trees, under which a more rapid and complete recovery would have been expected following rewatering. Rather, a drought response mechanism with some legacy effect was likely involved (Ruehr et al. 2019).

The measured water potentials in the soil–plant system can be analysed in light of information from the vulnerability curves to give insight into possible hydraulic limitations or damage. As we did not complete the full suite of measurements on Day 0, we consider Day 2 as a relatively ‘well‐watered’ benchmark. The predawn leaf water potentials on Day 2 were close to 0, sap flow rates were comparable to Days 0 and 1, and assimilation rates were also on the order of those measured under comparable conditions as part of gas exchange response curves (Section S3). However, VPD was somewhat higher on Day 2, so it is possible that there were some controls on plant water use that day. On Day 2, the midday leaf water potentials indicated a relatively small hydraulic margin as the leaf water potentials approached the stem $P_{50}$ of −1.5 MPa (the least negative $P_{50}$ in the plant). Despite this small hydraulic margin, it is unlikely that the plants experienced significant embolism over the course of the drydown. Although these midday leaf water potentials on Day 2 were close to the stem $P_{50}$ value, they were still less negative than the leaf $P_{50}$ value and they were also accompanied by high sap flow rates, such that the water potential in the stem at that time would be less negative than the leaves due to the water potential gradient necessary for water transport. The predawn leaf water potentials (when the leaves are presumably in equilibrium with the stem) did further decline over the course of the drydown, but they still remained less negative than both the stem $P_{50}$ and the leaf $P_{50}$ (−2.5 MPa). This suggests that neither the stem nor leaves reached water potentials where significant embolism would have occurred. The lack of significant xylem damage is further supported by the relatively rapid response to the rewatering, in which a small but clear pulse in sap flow occurred and leaf water potentials recovered close to 0 MPa within hours of rewatering (Section S17). Had significant hydraulic damage occurred, this would have likely hindered this water uptake into the sapwood resulting in a slower recovery of leaf water potentials (Brodribb et al. 2010).

Given the lack of evidence for hydraulic damage, an alternative hypothesis for the delayed recovery of plant fluxes is that the observed response and recovery were primarily driven by a hormonal/biochemical mechanism (McAdam 2023). The delay in sap flow recovery was mirrored by similar delays in the recovery of leaf gas exchange. Even at Day 15, the stomatal conductance values were still lower than they were at the beginning of the experiment and the assimilation rates were also lower, consistent with stomatal limitation of photosynthesis. Similar patterns of rapid recovery of plant water status but delayed recovery of gas exchange have been observed for other species and settings with various mechanisms proposed (Bi et al. 2023; Liang and Zhang 1999; Ruehr et al. 2019; Gallé, Haldimann, and Feller 2007; Creek et al. 2018; Torres‐Ruiz et al. 2015; Yao et al. 2021).

One plausible mechanistic explanation is that there was the synthesis of the hormone abscisic acid (ABA), which induces stomatal closure and inhibits growth as a means of limiting water losses (Tardieu and Davies 1993; Mittelheuser and Van Steveninck 1969; Raschke 1975; Davies et al. 1981; Nolan et al. 2017). Importantly, the effects of ABA can persist even after rehydration if there are still residually high concentrations of ABA (Brodribb and McAdam 2013; Belfiore et al. 2021; Hasan et al. 2021), and the effects can also include leaf senescence and abscission (Zhao et al. 2016; Kane and McAdam 2023). Simultaneous with the decoupling of sap flow and plant water potentials, there was also significant progression of leaf yellowing and shedding during the recovery period, supporting the hypothesised role of ABA, although the available data are not conclusive.

Other studies have also identified changes in the ethylene emission rate as another biochemical mechanism that can delay stomatal reopening and the recovery of gas exchange following drought (Bi et al. 2023; Yao et al. 2021). Carbon dynamics can also play a role in response and recovery, with the depletion of non‐structural carbohydrates under water stress potentially impacting repair and regrowth capacity after rewatering (Ruehr et al. 2019). However, with our measurements largely focused on water fluxes, it is hard to assess the extent to which changes in carbon metabolism may have played in the observed behaviour, especially given the short time frame of the observations. More broadly, better understanding these metabolic dynamics and their coupling with mechanisms controlling water fluxes remains an important area of research (Kannenberg and Phillips 2020; Galiano et al. 2017; Zeppel et al. 2019).

The combination of changes in leaf area and sustained stomatal closure would account for the delayed and incomplete recovery in sap flow. While our data do not enable us to partition the sap flow response into leaf area changes and stomatal closure, respectively, this would be potentially important for determining the long‐term recovery trajectory. The strength of this response is also significant in light of information about *P. trichocarpa* from the vulnerability curves, where it was seen to be relatively vulnerable overall and exhibited reverse vulnerability segmentation with stem xylem more vulnerable to cavitation than leaf xylem (Wilkening et al. 2023). It has been suggested that a strong ABA response to water deficits could be important for plants that are relatively vulnerable, since water‐potential‐induced closure of stomata alone is likely to result in low enough water potentials to still cause significant embolism in vulnerable plants (Brodribb and McAdam 2013). Additionally, while the vulnerability segmentation pattern of this species does not support the hypothesised ‘leaves as hydraulic fuses’ behaviour, it indicates an avenue by which this behaviour can occur, but through hormonal rather than hydraulic means.

More broadly, these results provide a case study of what will likely become an increasingly relevant scenario of plant behaviour during and after a moderate drought. While much attention has been directed towards understanding plant hydraulics as a driver of mortality under severe drought and significant efforts have been made to integrate this understanding into frameworks and models, less attention has been given to understanding how moderate droughts can potentially have a lasting impact on plant function and fluxes (McAdam 2023; Vilonen, Ross, and Smith 2022). There is a need for a better understanding of hormonal drought response mechanisms in natural systems, potential impacts on morphology and how these effects can scale up to the ecosystem scale. An important strategy for furthering understanding of the role of these varied physiological mechanisms will be collecting more comprehensive datasets. Through this exercise of qualitatively synthesising the data collected in this experiment, it was clear that the ability to support or reject certain hypotheses would have been far more limited if we had not been able to combine the information from different types of measurements.

### 4.2 Constraining Plant–Water Interactions

This experiment was unique in that it had a contained subsurface (i.e., pots), measured a number of different variables simultaneously and used dual isotope tracers. In analysing the data qualitatively, the value of multiple measurements was demonstrated as the data were able to provide evidence for testing hypotheses about different drivers and mechanisms. However, it is not necessarily practical or even possible to always achieve such a comprehensive set of measurements. To help provide more pragmatic takeaways about how to better constrain plant water dynamics, we can also use this dataset to consider the challenges and relative value of the different measurements, both quantitatively and qualitatively.

The varied mutual information values provide some quantitative insight into the ability of different measurements to reduce uncertainty in plant water dynamics during the experiment that is independent of any mechanistic frameworks for plant water use. We can first consider some of the variables or groups of variables which had higher mutual information values, suggesting that they might provide relatively better constraints. The physical environmental variables had relatively high mutual information values for the experimental period for both the sap flow and leaf water potentials. While the meteorological variables did not play a prominent role in our mechanistic analysis, the information value of the soil and meteorological variables makes sense given that they form boundary conditions for the water fluxes through the plant. For both predawn and midday leaf water potentials, the VPD and solar radiation had some of the highest mutual information values. However, it is worth noting that when only considering the sampling days there was some small variation in VPD and irradiation that was likely conflated with the soil moisture measurements in the mutual information analysis.

Of the physiological measurements, the daily minimum stem water content had the highest mutual information with sap flow over the entire experiment. Physically, the stem water content is closely related to sap flow as it is the integrated balance of root water uptake and transpiration water losses at the leaf. This close tie between sap flow and stem water content has also been observed in other species and settings and further emphasises calls that this process should be further incorporated into models of plant water use (Matheny et al. 2015, 2017). The higher mutual information of the minimum value versus the maximum value of stem water content is also likely due to the temporal nature of stem water content fluctuations, with the minimum typically occurring during the middle of the day when sap flow is at its peak and the maximum typically occurring at night when there is negligible transpiration. However, the minimum stem water content had relatively lower mutual information with both leaf water potentials, particularly for predawn leaf water potentials. Conversely to sap flow, this lesser ability of stem water content to constrain leaf water potentials could be because stem water content and leaf water potentials are less directly related mechanistically. The relationship between leaf water potentials and stem water content is mediated by plant water fluxes, rather than just a direct relationship.

The measurements of plant water sources from isotopic analysis, which were relatively consistent through the drydown and recovery periods, had notably low mutual information values for both sap flow and leaf water potentials, which varied across the time periods. This mirrored the somewhat challenging qualitative interpretation of the soil and plant isotopic measurements. The most prominent example of this is on Day 12, when Trees 2–6 all showed stem water that was more enriched in $\delta^{18}$O than any of the soil water sources. Taken with the sap flow data, which showed minimal sap flow between the rewatering and Day 12, the enriched $\delta^{18}$O measurements likely were indicative of stored water in the trees which still contained the enriched ^18^O tracer from the drydown. Other studies have observed similar decoupling of plant water and source water isotopes which were attributed to within‐plant storage and mixing (Brandes et al. 2007; Treydte et al. 2014). The isotopic signal of storage and mixing within plants has also been demonstrated with catchment‐scale modelling (Knighton et al. 2020). These data point towards the important role of water storage in plants, which can also provide challenges to the interpretation and utility of stable isotope measurements in some settings. However, while information about plant water sources did not necessarily provide strong direct constraints on plant water status and water fluxes, it could still potentially be valuable for improving model fidelity by providing an additional objective function within model frameworks. For the goal of trying to most efficiently constrain plant water fluxes or water status, water source measurements are likely less of a priority but could still be valuable if the resources are available for measuring them.

For constraining plant water status, considering both predawn and midday water potentials revealed some challenges. First, the mutual information between predawn and midday leaf water potentials was relatively low, such that the two different time points for measuring leaf water potential do not necessarily constrain each other very well. For both predawn and midday water potentials, there were a number of other measurements with which they shared higher mutual information, than compared to with one another. Theoretically, this can be explained by the fact that while predawn leaf water potentials can be thought of as an upper bound for the midday water potential, the actual deviation of midday water potential from that value can vary. This can also be seen in the generally lower mutual information values for midday leaf water potential compared to predawn water potential. The additional level of variability in midday water potential beyond the predawn water potential means it is likely more difficult to constrain. Given these temporal differences, it suggests that if there is a greater interest in daily minimum plant water potential (i.e., midday water potential), it would likely require greater constraints than compared to just daily maximum plant water potential (i.e., predawn water potential).

Across most of the measurements, a common pattern in the mutual information values is that the mutual information was generally lower during the recovery period than during the drydown period. While we did have fewer measurement days, and therefore smaller sample sizes for the recovery period than compared to the drydown period, these trends were also reflected in our qualitative assessments. In qualitatively trying to identify mechanisms, this phenomenon was observed as a decoupling of different measurements which had been coupled during the drydown. This highlights how certain dynamics are more difficult to constrain than others. From these results, plant water dynamics following drought are not only an increasingly relevant scenario, as discussed previously, but also one which is potentially even more difficult to constrain than compared to actively water‐limited conditions. Pragmatically, this suggests that post‐water stress periods are a scenario for which intensive measurement campaigns should be prioritised. It is worth noting, however, that while the decoupling can limit the constraints provided by any one measurement from an information theory perspective, the decoupling of measurements can be valuable from a mechanistic perspective. For example, the leaf water potential measurements had lower mutual information values with sap flow across the experimental period, such that it can be difficult to constrain plant water fluxes using plant water status and vice versa. However, we assessed qualitatively that the changing coupling between leaf water potential and transpiration is likely a result of the physiological mechanisms within the plant and, therefore, makes the observed decoupling valuable from a mechanistic standpoint, even if it means that the measurements are less valuable from the perspective of constraining plant behaviour. Additionally, given the design of the experiment, we only considered the mutual information between measurements taken on the same day. However, it could be interesting to examine the relationship between different measurements collected on subsequent days for datasets with greater temporal resolution.

### 4.3 Challenges and Opportunities Moving Forward

Even with the current level of data analysis, the experiment still points towards a number of broader challenges and opportunities for dealing with the high‐dimensional nature of soil–plant–atmosphere systems. First, the diversity and complexity of plant physiological responses to water limitations were evident in the response of the trees to the drydown and re‐watering. While plant hydraulics and embolism have received a lot of attention in terms of trying to understand and predict plant water stress, this experiment revealed the important role that morphological plasticity and hormonal responses play in mediating plant function and the fluxes of water and carbon. While not wholly unrelated to hydraulics since they play a role in moderating plant water status, they do have distinct mechanisms and dynamics, and their role in mediating ecosystem drought response, to this point, has been relatively unexplored. Connected to this more complicated role of physiology, the experiment also pointed towards some of the challenges of modelling and predicting plant responses to water limitations. With both the delayed response of sap flow and the assessment of water sources following rewatering, it was shown that there is the potential for ‘lags’ that are moderated by physiological processes. In this case, hormonal responses and plant water storage both created scenarios, where variables responded on different timescales to changing environmental conditions and there was decoupling between variables. This underscores the importance of bringing further physiological consideration into ecohydrological models to capture actual dynamics, as well as the necessity for further study of plant behaviour after water stress (Vilonen, Ross, and Smith 2022). Such investigations encompassing behaviour and mechanistic responses both during and after water stress will also help to interpret how different species may exhibit more resistant or resilient strategies for drought stress (Mitchell et al. 2016). Another challenge that was not included in this study but is increasingly relevant is the impact of compounding events. The impacts of water limitations on vegetation are increasingly being complicated by other global change events including pest infestation (Anderegg et al. 2015), heat stress (Ruehr et al. 2016) and increased atmospheric ${\;\text{CO}\;}_{2}$ levels (Birami et al. 2020; De Kauwe, Medlyn, and Tissue 2021), which make it even more difficult to disentangle drivers of vegetation behaviour.

Despite these challenges, this work points to more comprehensive datasets as a valuable opportunity to help address them. The experiment demonstrated various situations where it was possible to draw more advanced, and in some cases more accurate, conclusions with the combination of different variables. For example, the conflicting patterns in leaf water potentials and sap flow following the rewatering were difficult to interpret independently in terms of the degree and timing of plant recovery, but together they were able to point towards the hormonal mechanism discussed above. Working through the different hypotheses for drivers of the observed dynamics was greatly aided by the synthesis of the different measurements collected. Concurrent datasets like this one are also incredibly useful for calibrating and validating modelling frameworks that increasingly incorporate more complicated plant water dynamics (Feng 2020; Paschalis et al. 2020). While there is clear value in this as a goal, there are also smaller but more practical steps we can take to better constrain and understand the complex dynamics of plant–water interactions. These can include targeting more intensive data collection towards certain conditions which are more difficult to constrain (e.g., drought recovery) and prioritising types of measurements that better serve certain goals (i.e., mechanistic understanding vs. predictive constraints).

## Supporting information

Supplementary Information

## Data Availability

The data from this experiment are available at https://doi.org/10.5281/zenodo.10685215. Note that this dataset is the minimally processed dataset. The scripts for fully processing the data and producing the results and figures presented here are available at https://github.com/jvwilkening/Drydown_Expmt_Data_Analysis.
