# Supplementary material for: Mind the Data Gap: Using a Multi‐Measurement Synthesis for Identifying the Challenges and Opportunities in Studying Plant Drought Response and Recovery
Source: Plant Cell Environ. 2025 Jan 14;48(5):3673–90. doi: 10.1111/pce.15349 (PMC11963493; doi:10.1111/pce.15349)
Supplement: Supplementary file 1 — Supplementary Information [file PCE-48-3673-s001.pdf]

Supplementary Information

S1 Tracer isotopic compositions

Tree Number(s)	Application Day	Upper/Lower	$\delta^{18}O$ (‰)	δD (‰)
1	0	Lower	-13.77	359.1
2	0	Lower	-13.86	355.4
3	0	Lower	-12.70	355.0
4	0	Lower	-10.07	360.6
5	0	Lower	-10.58	355.8
6	0	Lower	-9.09	358.3
1	0	Upper	120.40	-77.8
2	0	Upper	88.08	-80.1
3	0	Upper	104.54	-79.5
4	0	Upper	108.58	-79.4
5	0	Upper	128.84	-78.7
6	0	Upper	120.00	-79.2
1,2,3	10	Upper	-14.95	348.9
4,5,6	10	Upper	-12.23	367.1

Table S1: Isotopic composition of tracers used throughout experiment

S2 Tracer trial data

Prior to the main experiment, the tracer application was tested on two additional trees (T1 and T2) to test distribution within the soil and the time to uptake by the trees. The trees were smaller individuals of the same species, but were in the same containers as the trees used in the main experiment. Starting on June 3rd, 2021, transpiration was collected from each tree by attaching plastic bags to portions of branches in the morning and collecting the condensed transpiration that accumulated over the course of the day (attached a minimum of six hours). On June 8th, 2021, D-enriched tracers were applied to each tree. For Tree T1, 5.5 L of a tracer with isotopic signature -12.91‰ δ^{18}O and 348.2‰ δD was applied to the surface, and later that day 8.3 L of unlabelled tap water ($\approx$ -12.23‰ δ^{18}O and -88.0‰ δD) was injected into the middle of the container following the same procedure as in the main experiment. For Tree T2, 5.5 L of unlabelled tap water was applied to the surface, and later that day 8.3 L of a tracer with isotopic signature -14.29‰ δ^{18}O and 355.3‰ δD was injected into the middle of the container following the same procedure as the main experiment. The following day, soil samples from different depths were taken using the same procedure as the main experiment. Profiles of soil samples were taken from three different locations along the perimeter of the container for each tree. Water was extracted and the isotopic signature was analyzed using the same procedure as for the soil samples from the main experiment. The soil water isotopic profiles are shown in Figure S1. Transpiration samples were collected for the 15 days following the application of the tracers and analyzed using IRIS. The transpiration time series are shown in Figure S2. Based on the results of these trials, the containers in the main experiments were placed on pieces of wood to better ensure free drainage at the bottom of the container, but the procedures were otherwise consistent.

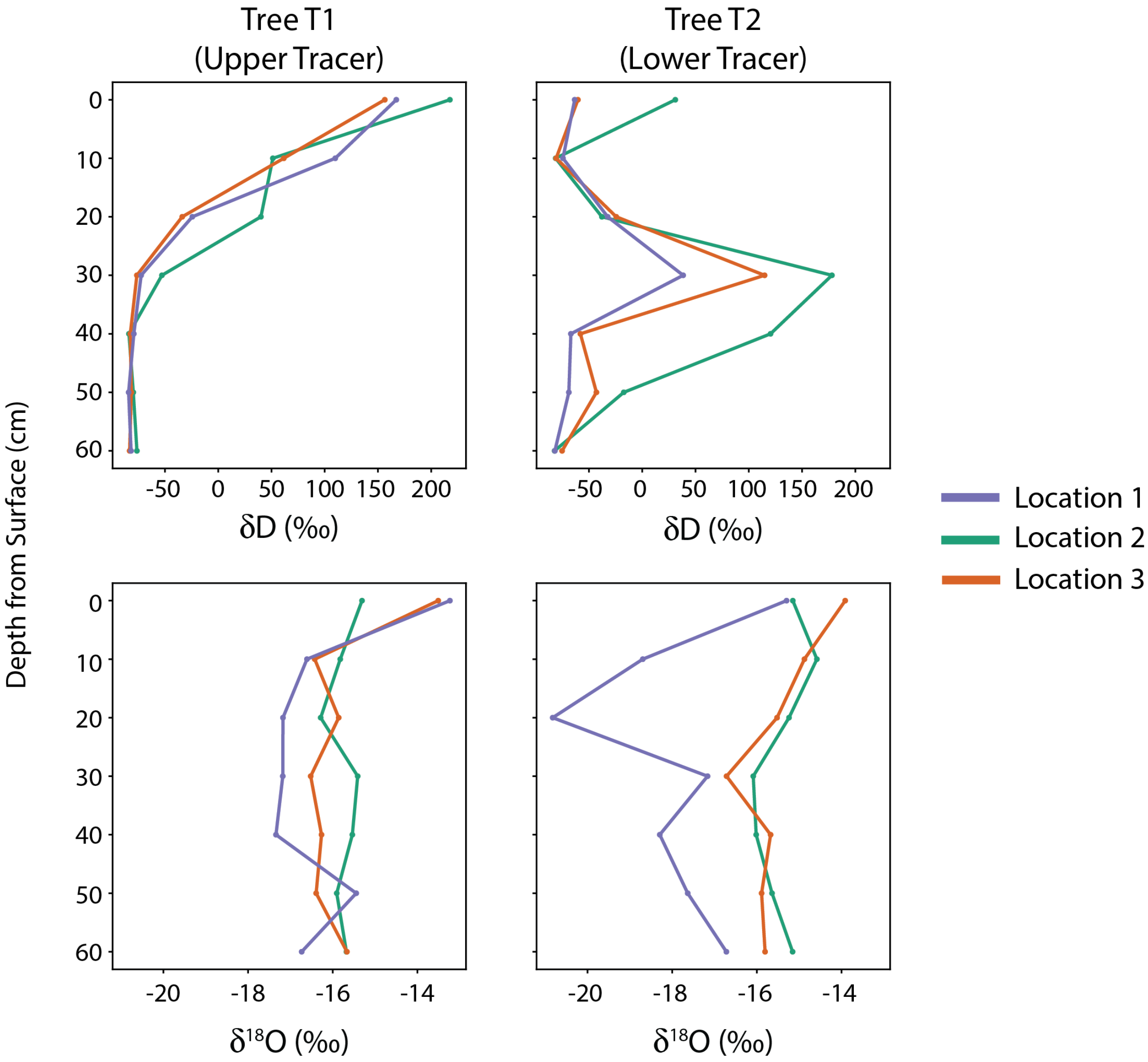

Figure S1: The soil isotope profiles are shown for Tree T1 (left column), which had a D tracer applied at surface, and Tree T2 (right column), which had D tracer injected in the middle of the container. The upper row of plots show the δD depth profiles and the lower row of plots show the δ^{18}O depth profiles. For each tree, three soil profiles were sampled at different locations around the circumference of the container, which are denoted with the different colors.

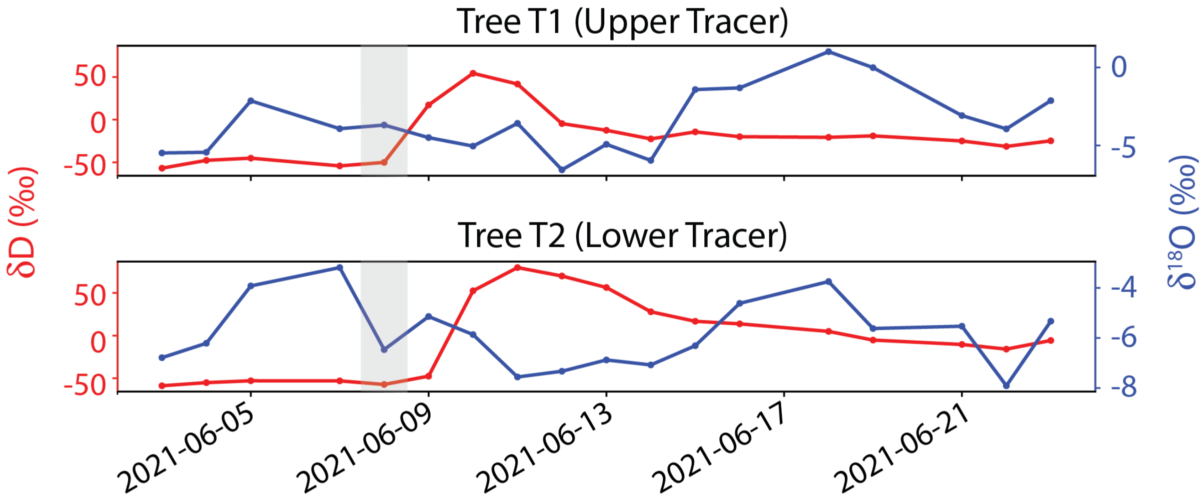

Figure S2: Time series of transpired water isotopic composition for Trees T1 (upper D tracer) and T2 (lower D tracer) during the tracer trial. The D tracers were added on June 8th, 2021, which is denoted with the gray box. Data shown for δD in red (lefthand y-axis) and δ^{18}O in blue (righthand y-axis).

S3 Gas exchange response curves

Prior to the start of the experiment, light and CO_2 (A/C_i) response curves were measured for the experimental trees using a portable photosynthesis system (LI-COR LI-6800). During the measurements, the chamber was set to 70% relative humidity and a leaf temperature of 27°C. During the light response curves, assimilation was measure for light source levels starting at 1800 µmol m^{-2} s^{-2} down to 0 µmol m^{-2} s^{-2} in 200 µmol m^{-2} s^{-2} increments. The chamber CO_2 concentration was set to 400 µmol mol^{-1}. During the A/C_i, the measurements of assimilation started at a C_i approximately 330 µmol mol^{-1} which was then stepped downwards to approximately 25 µmol mol^{-1} and then increased in increments up to approximately 1050 µmol mol^{-1}. During the A/C_i curves, the light source was set to 1500 µmol m^{-2} s^{-2}.

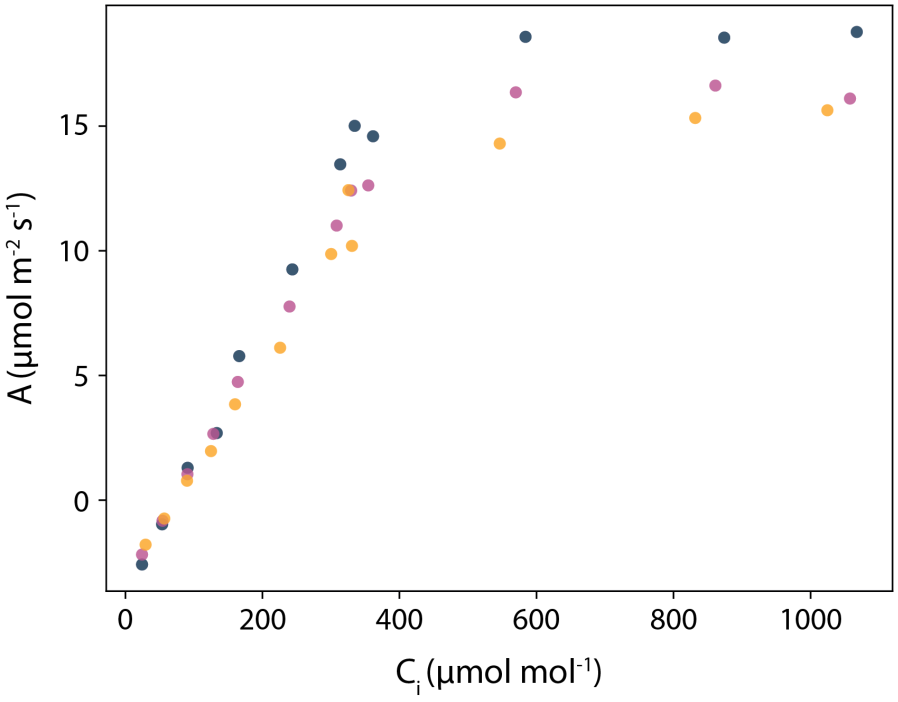

Figure S3: Three CO_2 response curves were measured prior to the experiment. The colors of the points represent different leaves.

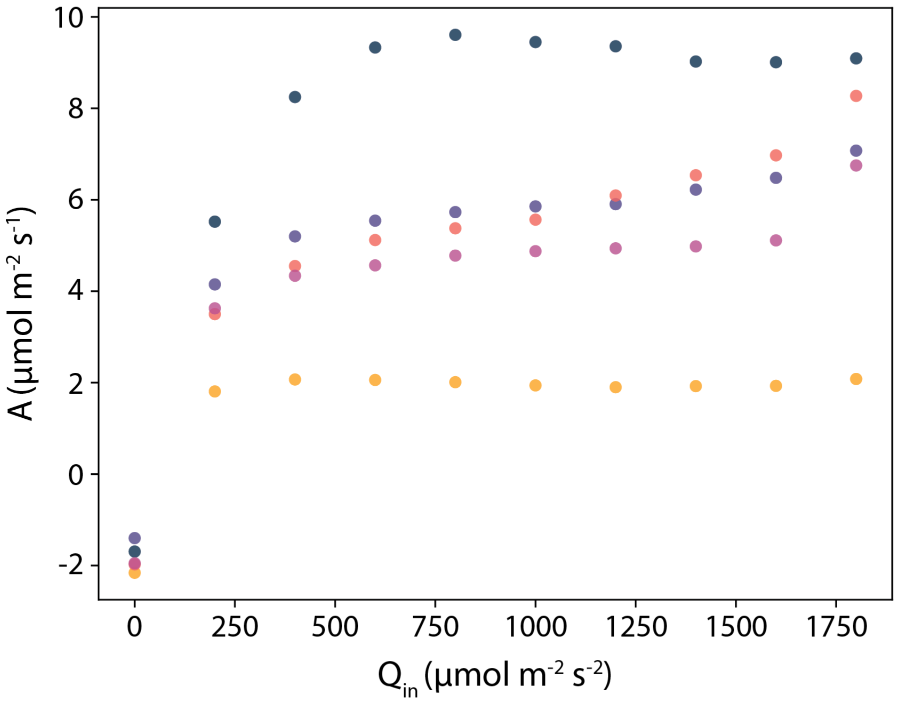

Figure S4: Five light response curves were measured prior to the experiment. The colors of the points represent different leaves.

S4 Tree morphological data

Tree Number	Tree Height (m)	Tree Diameter (cm)	Final Leaf Area (m^2)
1	3.65	4.07	2.273
2	3.29	2.88	-
3	3.31	3.13	-
4	2.68	2.99	1.336
5	3.27	3.40	1.002
6	3.15	3.05	-

Table S2: Measured tree morphological data. Final leaf area was only measured for the trees which were cut at the end of the experiment.

S5 Root mass distributions

The depth profiles of recovered root masses from the subsamples for the three sampled trees are shown in Figure S5. Overall, the root mass measurements were variable across the depths, with no obvious trends with depth across the trees. In general, Tree 1 had relatively lower root masses than the other two trees. Other morphological data are reported in Supplementary Table S2.

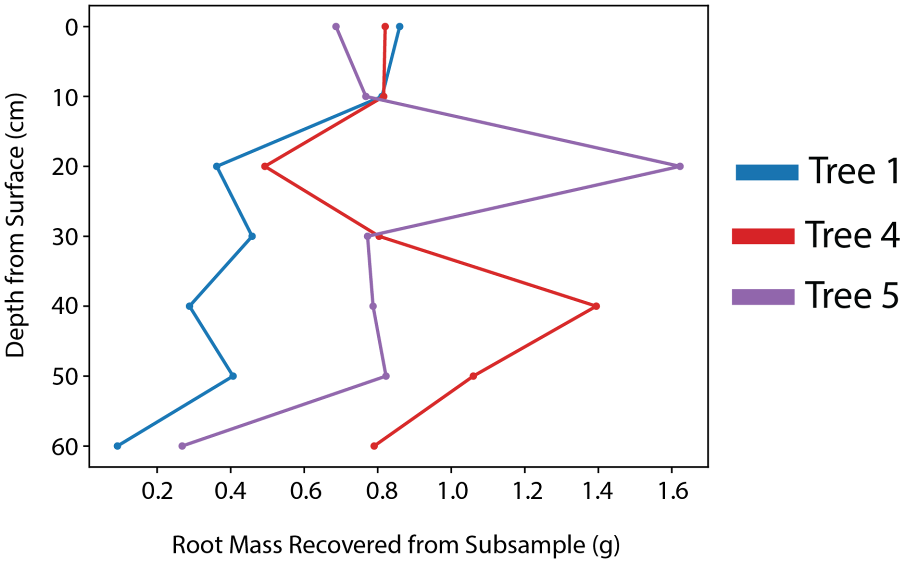

Figure S5: The recovered and dried root masses for each soil depth subsample are shown, with the line/point colors corresponding to individual trees.

S6 Soil water retention curve

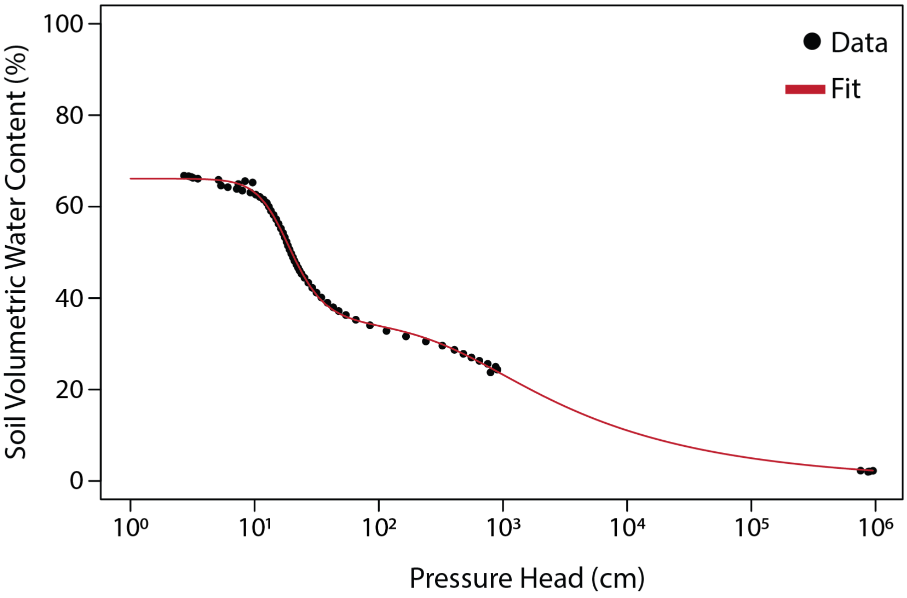

Figure S6: Soil water retention curve for the first soil replicate. The black dots show measurements and the red curve shows the fitted bimodal van Genuchten curve.

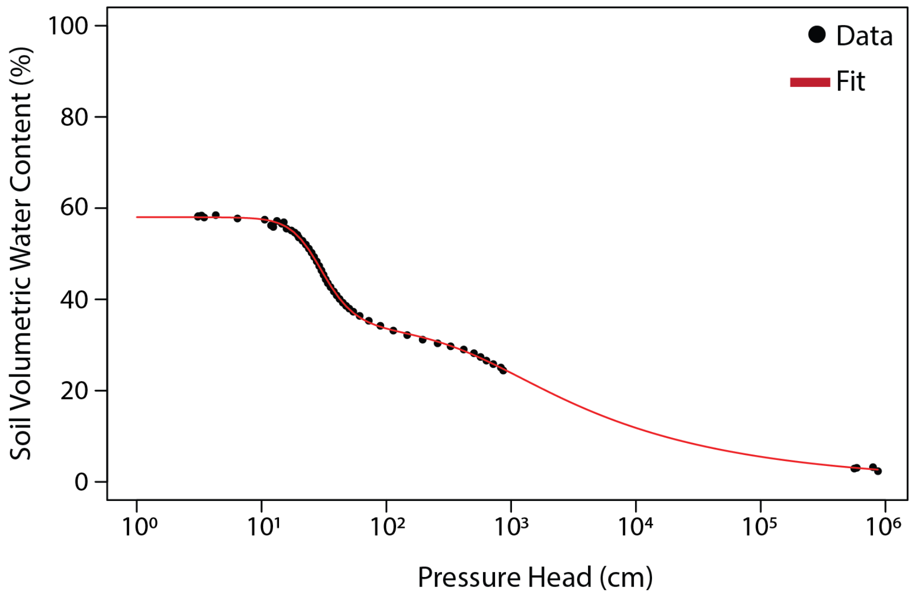

Figure S7: Soil water retention curve for the second soil replicate. The black dots show measurements and the red curve shows the fitted bimodal van Genuchten curve.

Replicate	θ_s	θ_r	α	n	ω_2	α_2	n_2
1	0.661	4.06×10^{-16}	1.347	0.00275	0.470	0.058	3.573
2	0.580	5.63×10^{-16}	1.333	0.00236	0.413	0.036	3.729

Table S3: Fitted parameter values for each replicate for the bimodal van Genuchten equation

S7 Transpiration isotope data

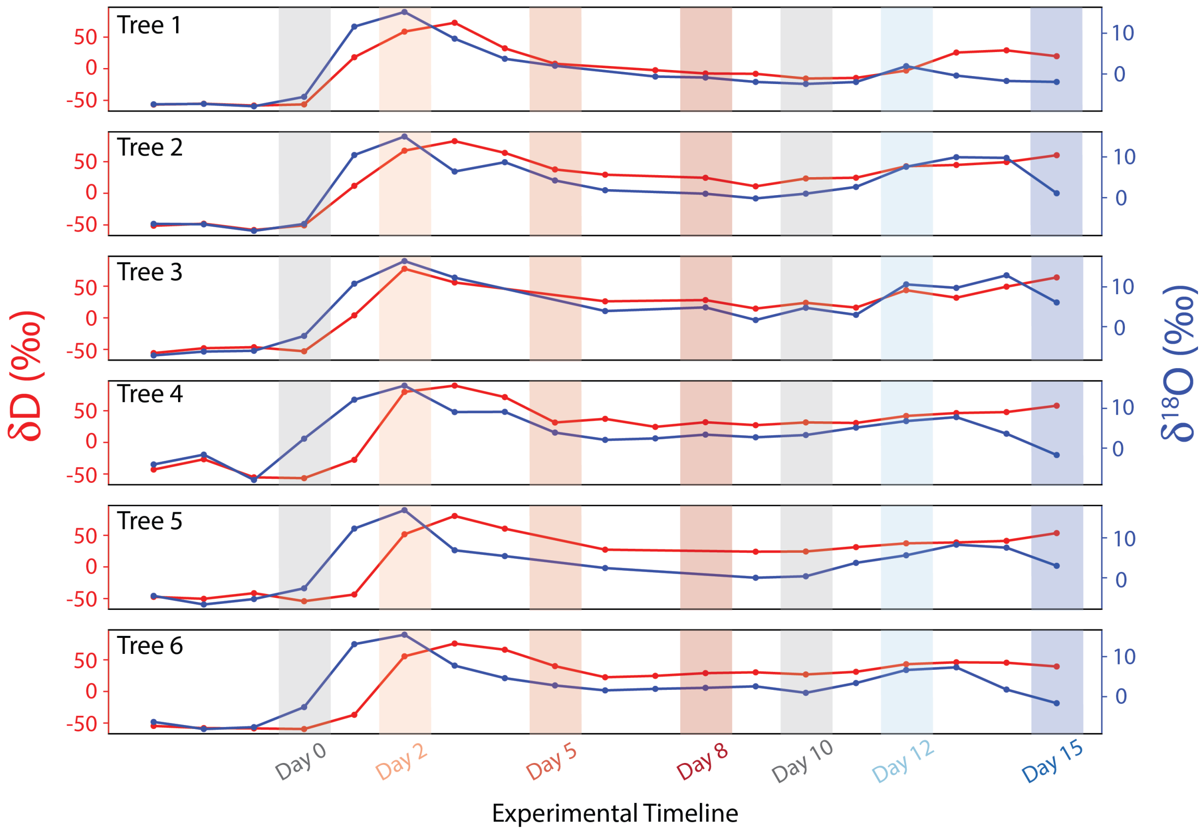

Figure S8: Time series of transpired water isotopic composition for all six trees over the course of the experiment, with sampling days shaded with colors and tracer addition days shaded in gray. Data shown for δD in red (lefthand y-axis) and δ^{18}O in blue (righthand y-axis).

S8 IRIS measurement validation

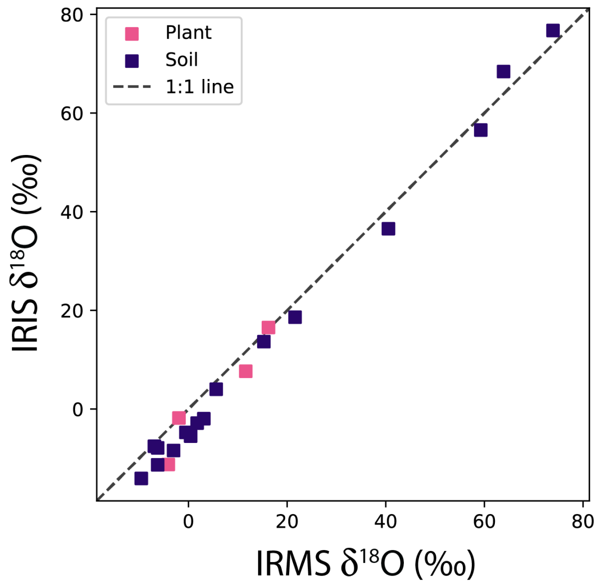

Figure S9: A subset of the soil (dark blue) and plant (magenta) water samples were analyzed for δ^{18}O using both IRMS (x-axis) and IRIS (y-axis).

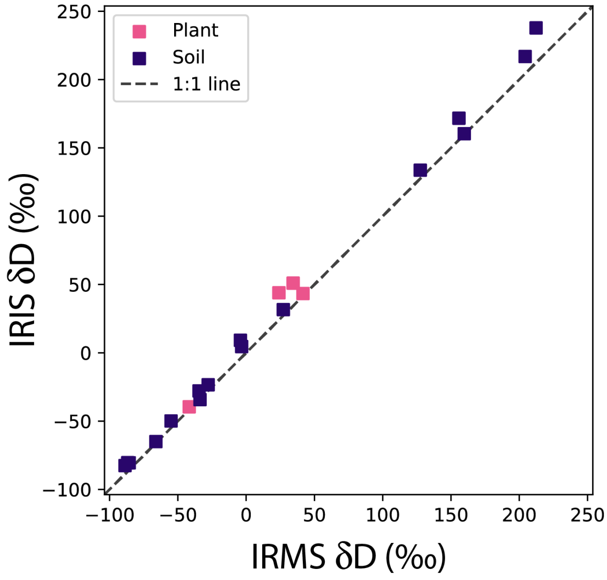

Figure S10: A subset of the soil (dark blue) and plant (magenta) water samples were analyzed for δD using both IRMS (x-axis) and IRIS (y-axis).

S9 Isotope summary calculations

To summarize the isotopic water source measurements for the mutual information analysis, the soil and plant water isotopic compositions were used to calculate a mean water uptake depth for each tree for each of the sampling days. The plant water δD and δ^{18}O are assumed to represent the mixture of waters from the soil layers, with no fractionation during root water uptake. Mathematically, this is described with the following equations:

$$\delta_{plant} = \sum_{i=1}^{n} f_i \delta_i \tag{S1}$$

where δ_{plant} is the measured isotopic signature of composition of the plant and is equal to the sum of the products of the fractional uptake f_i and soil layer isotopic composition δ_i for n soil layers, where:

$$1 = \sum_{i=1}^{n} f_i \tag{S2}$$

The soil column was divided into six 10 cm layers (0-10 cm, 10-20 cm, 20-30 cm, 30-40 cm, 40-50 cm, 50-60 cm), with the isotopic composition for each layer taken as the mean of the measurements for the respective bounds of the layer (e.g. the uppermost 10 cm layer is taken as the mean of the samples from 0 cm and 10 cm). The system of equations created by Equation S1 for both δD and δ^{18}O and Equation S2 is solved using optimization routines in the SciPy library of Python to find the fractional contribution (f) of each soil layer. The mean uptake depth is then calculated as:

$$MeanDepth = \sum_{i=1}^{n} f_i d_i \tag{S3}$$

where d_i is the depth from the surface of the soil for the midpoint of the soil layer.

S10 Meteorological data time series

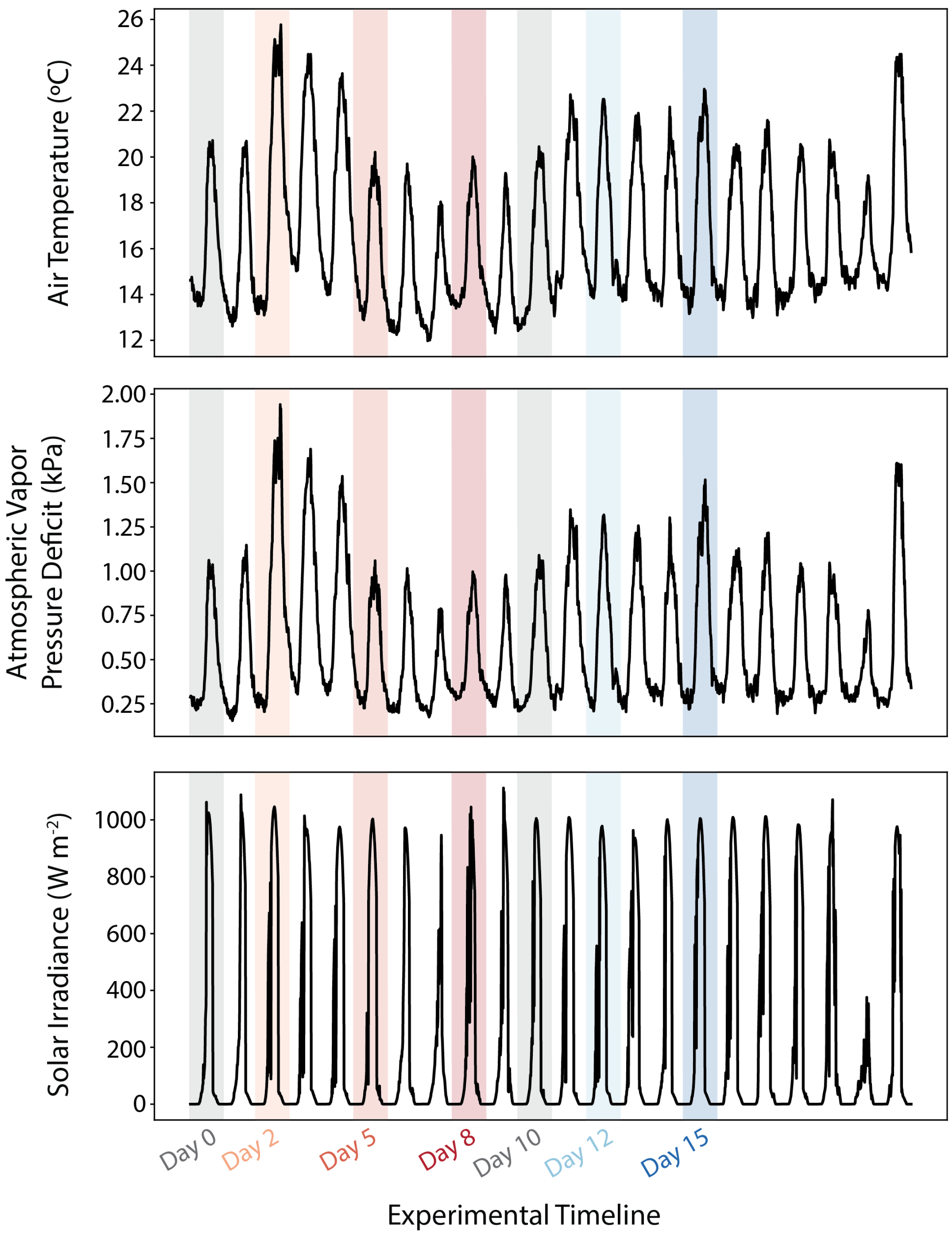

Figure S11: Meteorological data over the course of the experiment are shown with air temperature in the top panel, atmospheric vapor pressure deficit in the middle panel, and solar irradiance in the bottom panel. The atmospheric vapor pressure deficit values are calculated from the measurements of air temperature and relative humidity. Shaded bars highlight the sampling (colored) and watering (gray) occurred.

S11 Soil water potential

We do note some caution in the interpretation of the soil water potential values, particularly on the dry end of the spectrum. The manufacturer of the probe used to measure soil water content state the instrument has an accuracy of +/- 3% VWC, and it is further possible that there was spatial heterogeneity in soil moisture that our measurement approach was unable to capture. While this is more or less negligible in wetter soils, in drier soils this can translate to large differences in soil water potential, due to the highly non-linear nature of the soil water retention curve. For example an instrument reading

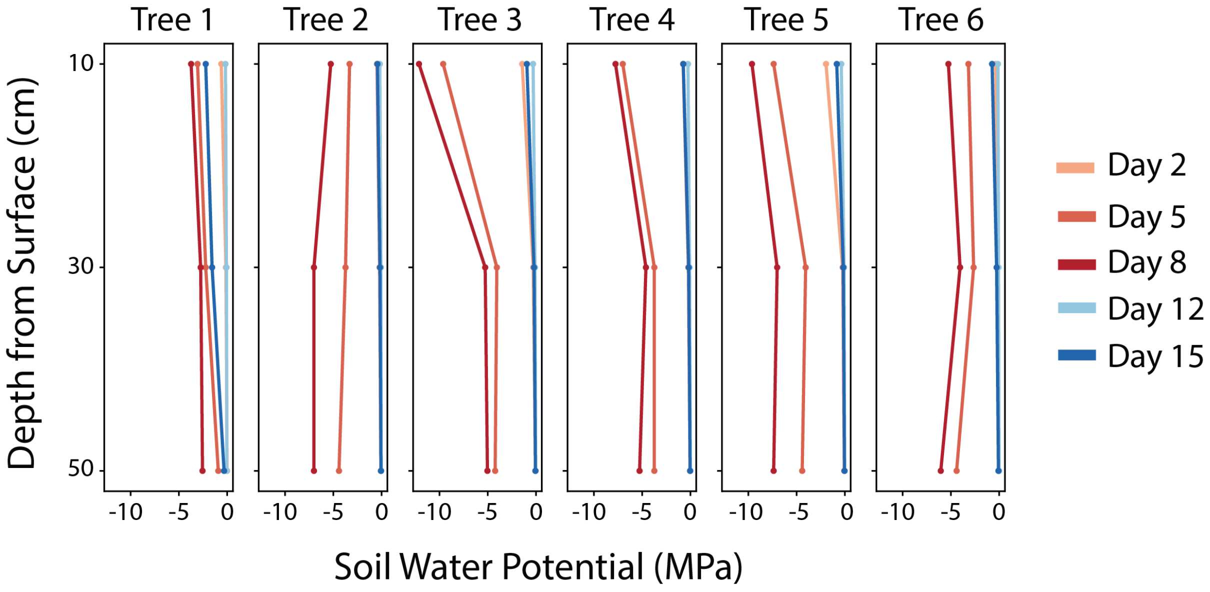

Figure S12: The soil water potentials (MPa) at different depths across the sampling days are shown across the panels for each tree. Measurements of soil volumetric water content (V_{water}/V_{soil}, %) were made at depths of 10, 30, and 50 cm below the surface, and converted to water potential using the measured soil water retention curve. The different sampling days are denoted with different line/point colors, with the drydown period in shades of red and the recovery period in shades of blue.

of 8% VWC in the experimental soil (within the range of soil moisture values recorded on Days 5 and 8) would correspond to a range of soil water potentials traversing an order of magnitude (-11.42 to -1.11 MPa) when the instrument error is translated into the soil water potential.

S12 Full sap flow time series

The full time series for total sap flow and stem water content for each tree are shown in Figures S13 and S14, respectively. During the initial days of the drydown (Days 0-3), the highest peaks of sap flow were observed and were accompanied by large diurnal variations in stem water content. Days 4-5 showed large declines in daily sap flow rates across all trees and a dampening of the diurnal variation in stem water content. The midday stem water content drawdown was less pronounced and overnight the stem water content did not fully return to the same values observed in the preceding days. Days 6-10 showed minimal sap flow and only small fluctuations in stem water content. Following rewatering on Day 10, a small spike in sap flow was observed, as well as an increase in stem water content. Between Days 11-15, there was a slow increase in sap flow rates and the stem water content variations slowly return to patterns similar to those observed at the beginning of the experiment. However, even by Day 15 the sap flow rates remained lower than those observed at the beginning of the experiment. In the days following the conclusion of the experimental period, these patterns persist.

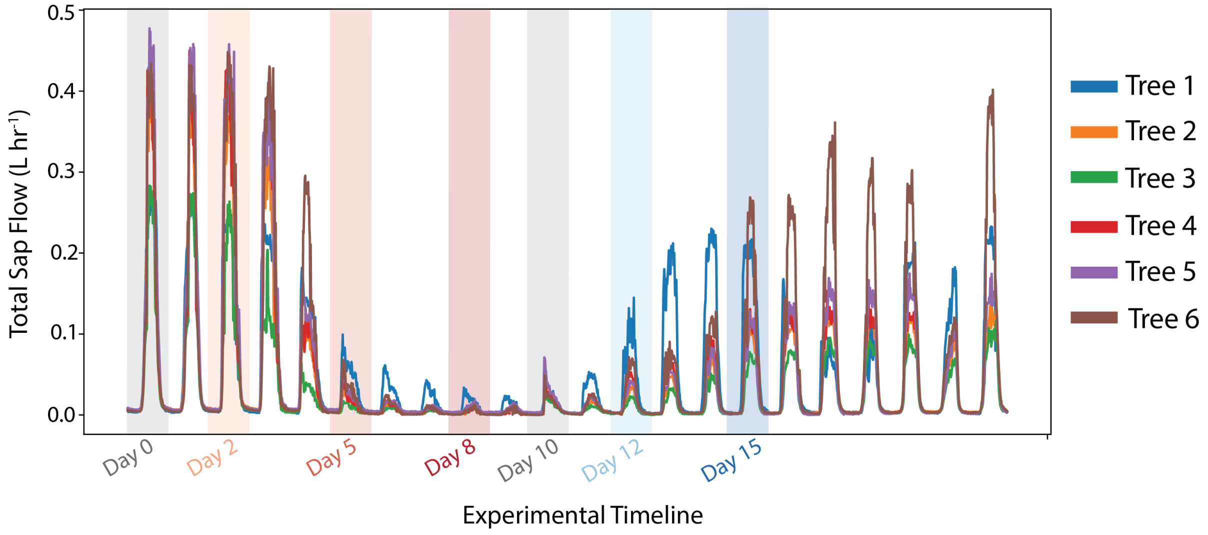

Figure S13: Total sap flow rates (L hr^{-1}) are shown for the duration of the experiment. Shaded bars highlight the sampling (colored) and watering (gray) occurred. The individual trees are presented with different color lines.

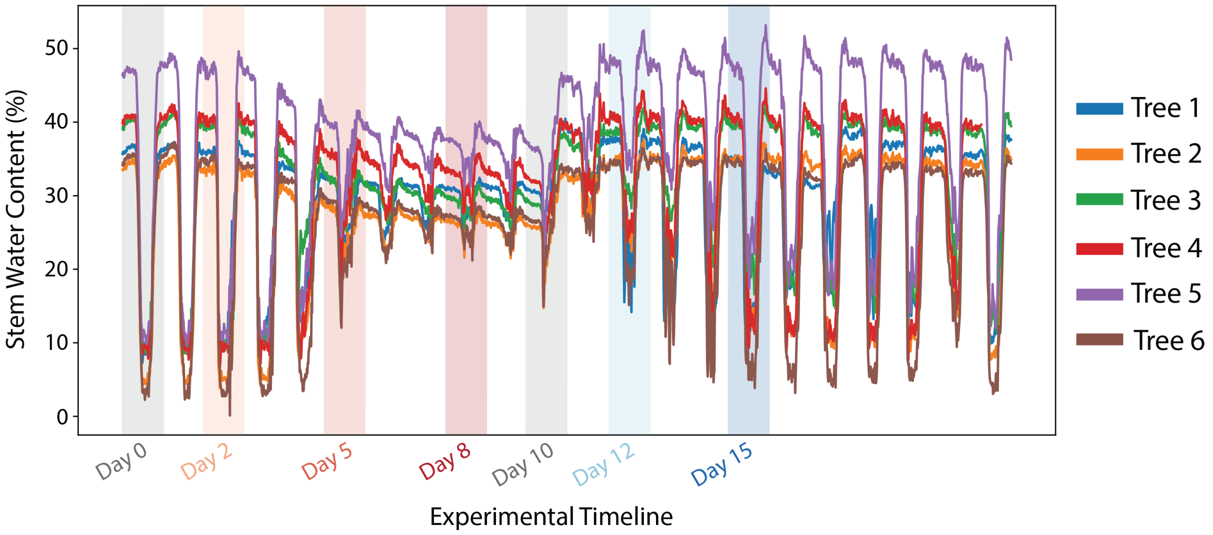

Figure S14: Stem water content measurements (%) are shown for the duration of the experiment. Shaded bars highlight the sampling (colored) and watering (gray) occurred. The individual trees are presented with different color lines.

S13 A from gas exchange measurements

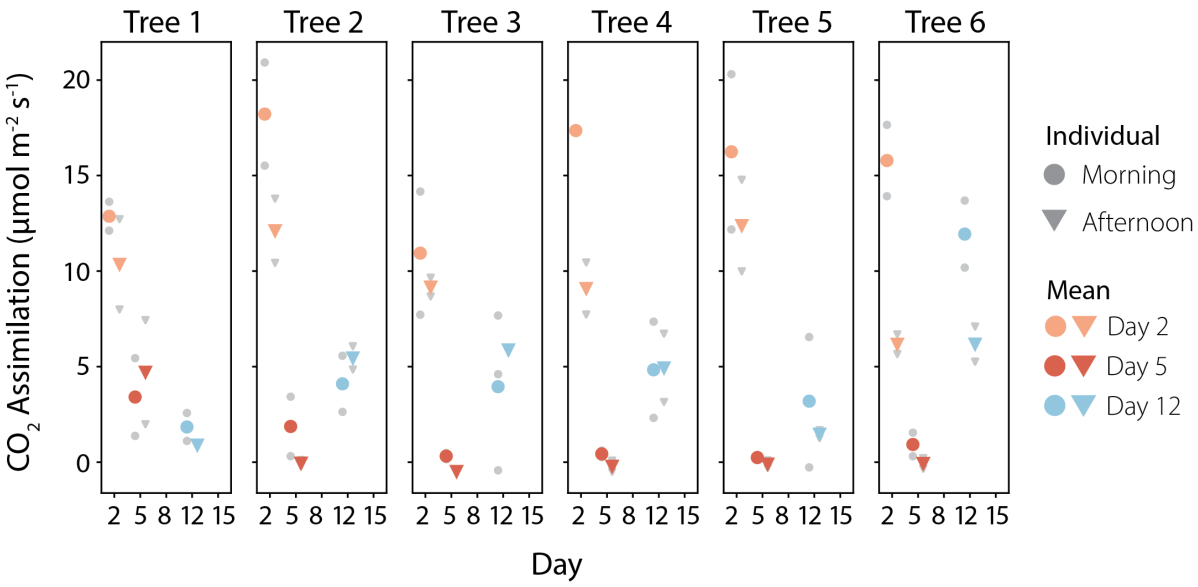

Figure S15: CO_2 assimilation rates (µmol m^{-2} s^{-1}) from the sampling days for each tree are shown across the panels. Individual measurements are shown as gray points, with the morning measurements (10:15-11:15) shown as circles and the afternoon measurements (14:30-15:30) shown as triangles. The means for each day and sampling time are shown with colored points.

S14 Comparison g_s and A from exchange measurements

The relationship between assimilation rate and stomatal conductance is shown for the experimental data in Figure S16. On Day 2 between the morning and afternoon measurements, the trees show a relatively greater proportional decline in stomatal conductance than in assimilation rate. For example, one of the afternoon measurements for Tree 1 shows a similar assimilation rate as compared to the morning measurements, but at a much lower stomatal conductance. The remaining measurements for all the trees show a relatively linear relationship between stomatal conductance and assimilation.

S15 Soil water isotope profiles

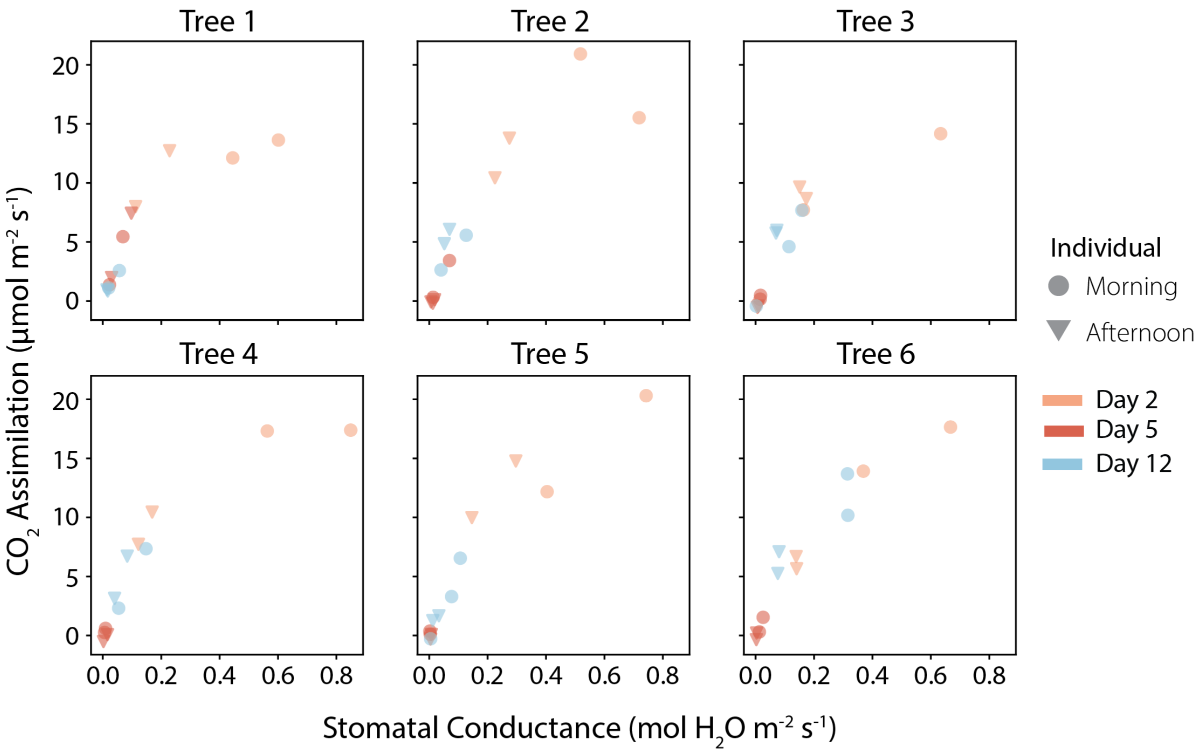

Figure S16: Here the individual measurements of stomatal conductance (mol H_2O m^{-2} s^{-1}) and CO_2 assimilation rates (µmol m^{-2} s^{-1}) are plotted against one another. The morning measurements (10:15-11:15) are shown as circles and the afternoon measurements (14:30-15:30) are shown as triangles. The colors correspond to the day the measurement was recorded.

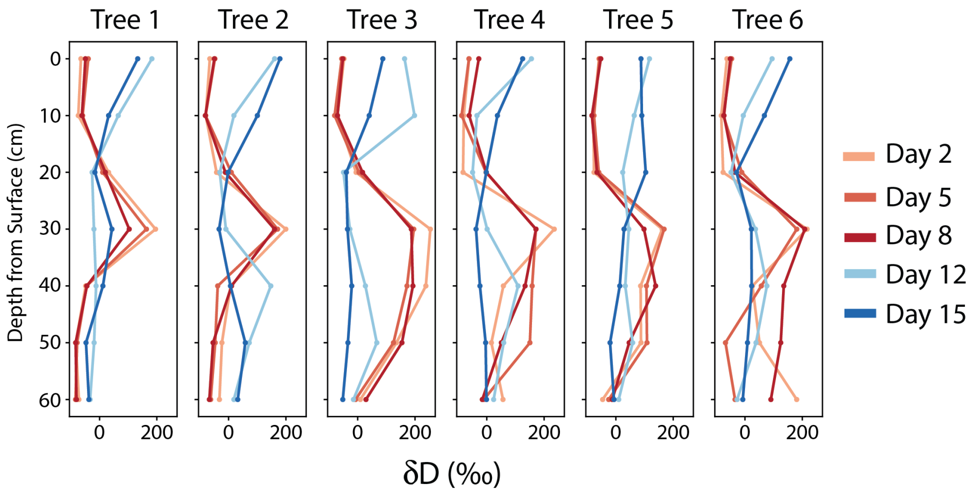

Figure S17: The depth profiles of δD for the soil water samples for each tree are shown. The colors correspond to the different sampling days.

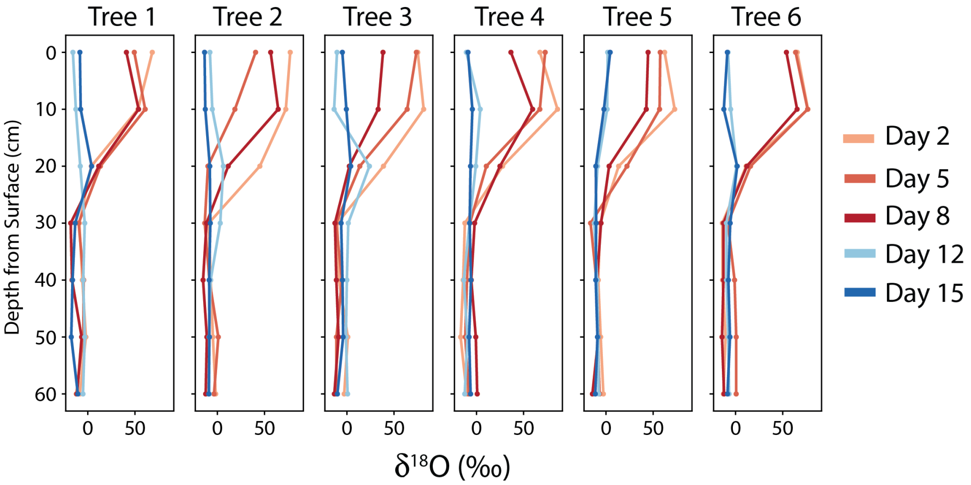

Figure S18: The depth profiles of δ^{18}O for the soil water samples for each tree are shown. The colors correspond to the different sampling days.

S16 Mutual information values

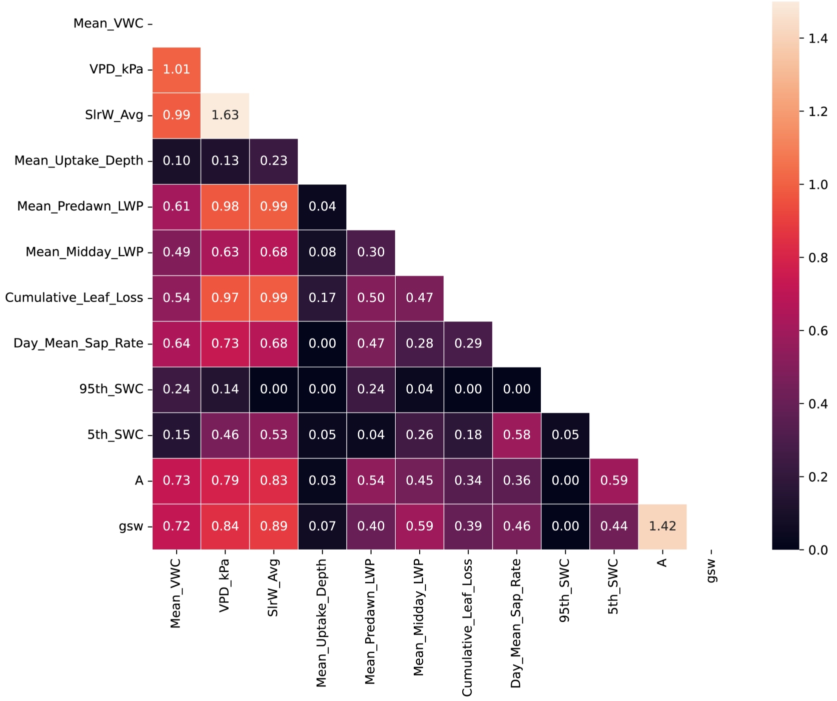

Figure S19: Pairwise mutual information values during the entire experimental period.

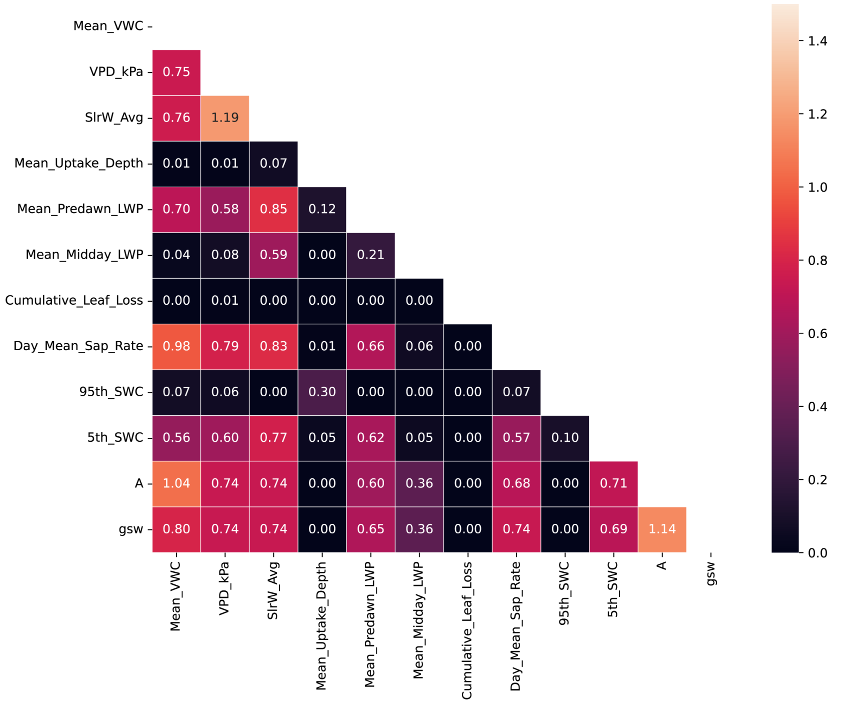

Figure S20: Pairwise mutual information values during the drydown period.

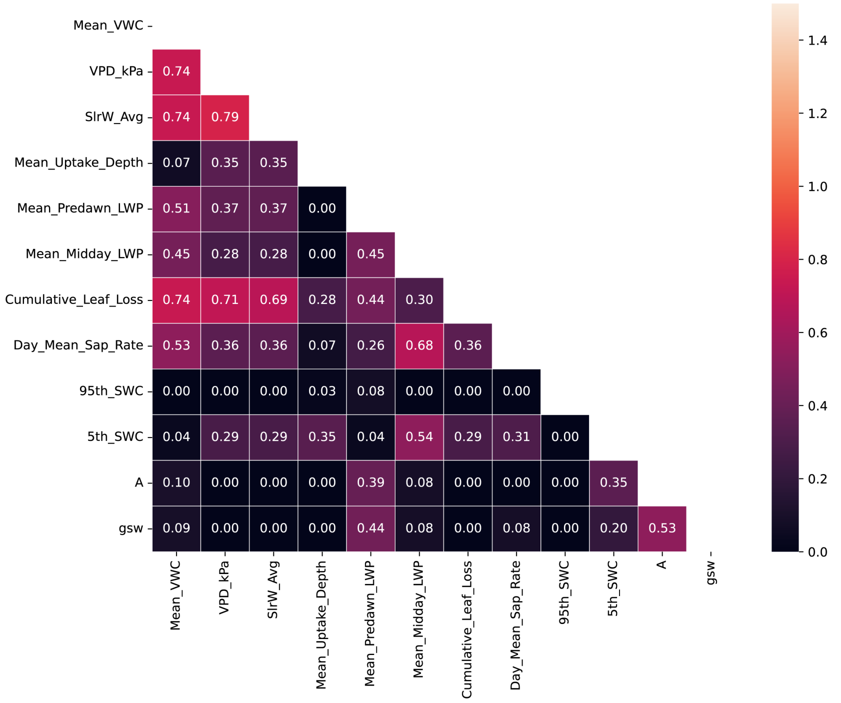

Figure S21: Pairwise mutual information values during the recovery period.

S17 Additional measurements during rewatering (Day 10)

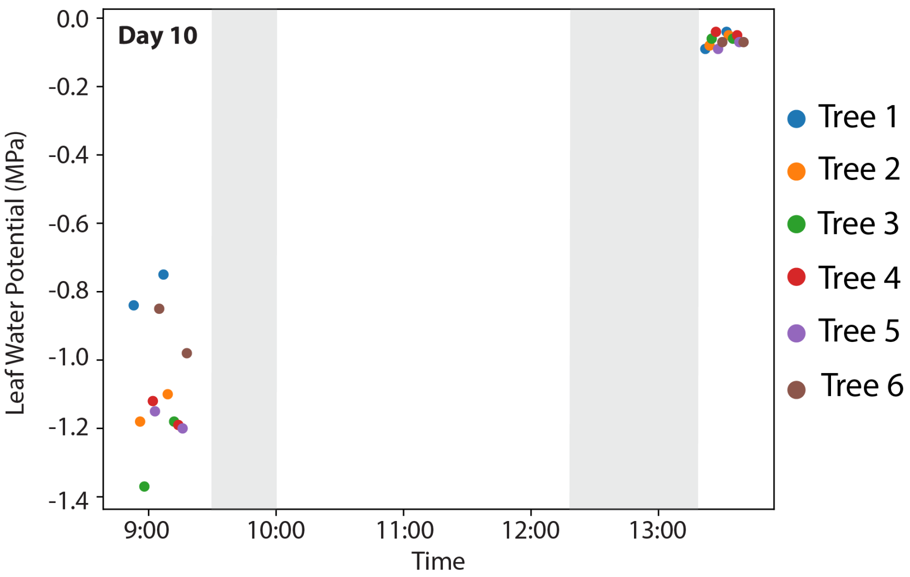

Figure S22: Two replicates of leaf water potentials for each tree (denoted by colors) were also measured before and after the rewatering on Day 10. The gray shaded boxes denote the time windows in which the two stages in which the rewatering was done. During the first stage, tap water was used to saturate the soil. Later in the afternoon, the surface δD tracer was added.

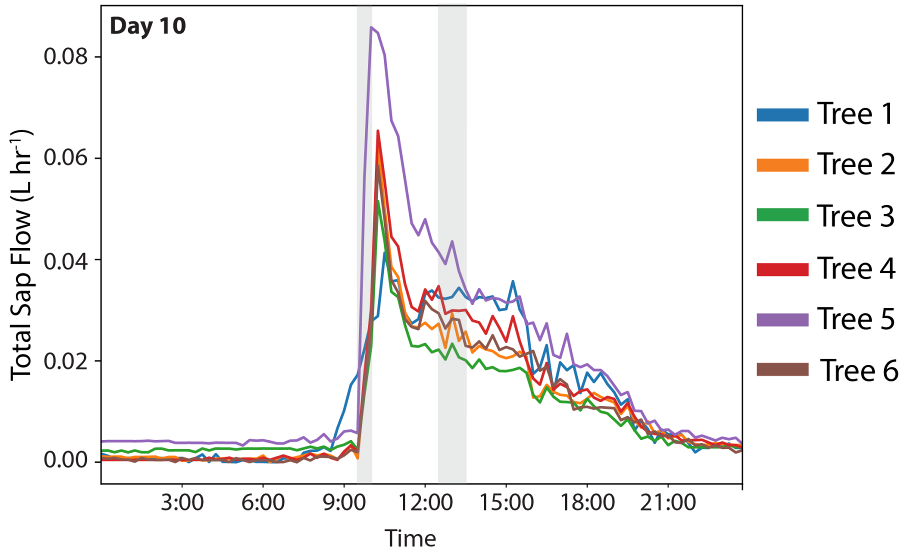

Figure S23: A detailed view of the sap flow measurements on Day 10 is shown here, with the individual trees denoted with different colors. Note that the y-axis scale here is much smaller than in Figure S13. The gray shaded boxes denote the time windows in which the two stages in which the rewatering was done. During the first stage, tap water was used to saturate the soil. Later in the afternoon, the surface δD tracer was added.
